# Metabolomics Revealed the Differential Metabolites of Different Broomcorn Millet Varieties in Shanxi

**DOI:** 10.1002/fsn3.70902

**Published:** 2025-09-03

**Authors:** Chao Jiang, Jiao Mao, Xiaoqiang Song, Hai Li, Xiaoning Cao

**Affiliations:** ^1^ Institute of Crops in Cold Regions Shanxi Agricultural University Datong China; ^2^ Center for Agricultural Genetic Resources Research Shanxi Agricultural University Taiyuan China

**Keywords:** amino acids, broomcorn millet, flavonoids, metabolites, phenolic acids

## Abstract

Broomcorn millet is a kind of coarse grain crop that is beneficial to health. However, there is still a lack of systematic information support for metabolic research on its nutritional value. This study used widely targeted metabolomics to compare different broomcorn millet varieties (Yanshu No. 13, Yanshu No. 16, Jinshu No. 9, Jinshu No. 15, and Pingshu No. 9). A total of 1119 metabolites were identified among different varieties of millet, including 16 major categories such as phenols, alkaloids, and phenylpropanoids. The results showed that the main differential pathways among different broomcorn millet varieties included tryptophan metabolism, purine metabolism, and phenylalanine metabolism. The grain color among different varieties of broomcorn millet is related to β‐carotene, glutathione, naringenin, and tryptophan metabolism. The abundance of various antioxidant compounds in colored broomcorn millet is higher than that in JSNO15. The accumulation of phenolic acids, flavonoids, and amino acids in different varieties of broomcorn millet varies significantly, which may be one of the reasons for the differences in broomcorn millet varieties. This study provides a theoretical basis for varietal breeding of broomcorn millet and the promotion of efficient utilization of broomcorn millet resources.

## Graphical abstract

A total of 1119 metabolites were identified among different varieties of millet, including 16 major categories such as phenols, alkaloids, and phenylpropanoids. The main pathways of differences among different varieties of millet include tryptophan metabolism, purine metabolism, phenylalanine metabolism, etc. The grain color correlates with β‐carotene, glutathione, naringenin, and tryptophan metabolism. Colored millet has a higher abundance of various antioxidants than JSNO15. Differences in phenolic acids, flavonoids, and amino acids contribute to millet variety variations.

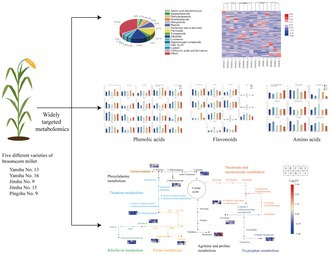

## 1 Introduction

Broomcorn millet ( *Panicum miliaceum* L.) originated in China (Lu et al. 2009). It is rich in proteins and minerals (Das et al. 2019), and has characteristics such as a short growth period, drought resistance, tolerance (Diao 2017), salt tolerance, and high water use efficiency (Yuan et al. 2021). The total planting area of broomcorn millet in China has remained stable at 800,000 to 1,000,000 ha, mainly distributed in areas with poor soil and an arid climate (Gong et al. 2018), or arid and semi‐arid regions (Wang et al. 2024). In addition, broomcorn millet does not contain gluten, which makes it very suitable for people with celiac disease to consume (Das et al. 2019). Compared with most other grains, broomcorn millet has a higher content of protein, vitamins, and various minerals (Li, Zhao, et al. 2021; Li, Wen, et al. 2021). As a crop that can provide people with healthier diets, broomcorn millet is attracting more and more people's close attention.

Broomcorn millet is not only rich in essential nutrients, but its unique bioactive components also endow it with significant health value (Liang and Liang 2019). Xiong et al. (2022) demonstrated through metabolomics that anthocyanins, phenols, and flavonoids are the main antioxidant components in purple glutinous rice and can be used in the food industry. Phenolic acids and flavonoids, as phenolic compounds, are present in broomcorn millet. These substances, due to their antioxidant activity, can help the human body eliminate free radicals and reduce the damage caused by oxidative stress (Liang and Liang 2019). These phenolic compounds have a regulatory effect on the intestinal microecology, selectively promoting the proliferation of probiotics while inhibiting the growth of pathogenic bacteria, thereby optimizing the structure of the intestinal flora (Sleem et al. 2024; Nicolás‐García et al. 2021). Furthermore, certain components in broomcorn millet may have anti‐inflammatory, anti‐tumor, and hypoglycemic effects (Udeh et al. 2017; Devi et al. 2014). Broomcorn millet protein can effectively increase high‐density lipoprotein cholesterol levels (Park et al. 2008), playing a role in protecting cardiovascular health (Oram and Heinecke 2005). These bioactivities provide a scientific basis for broomcorn millet as a functional food. Broomcorn millet is rich in phenolic acids and flavonoids, and there are significant differences in the composition and content of phenolic substances among different varieties of broomcorn millet (Chandrasekara et al. 2012; Chandrasekara and Shahidi 2010, 2011).

China's broomcorn millet is rich in genetic variations. Different varieties vary significantly in growth characteristics, stress resistance, and yield, which are of great research value. Li et al. found that the differences in the antioxidant capacity of broomcorn millet varieties were partly attributed to the changes in the content of phenolic acids and flavonoids, and the accumulation of these components varied with the color of the varieties (Li, Zhao, et al. 2021; Li, Wen, et al. 2021). Bai Lu constructed an F2 isolated population using yellow broomcorn millet I and 2016106I and conducted transcriptome sequencing (Bai 2022). Bai Lu et al. found that the formation of the color of broomcorn millet grains is closely related to the flavonoid biosynthesis pathway, among which cinnamaldehyde dehydrogenase and cinnamoyl‐CoA are the key functional genes regulating this trait (Bai 2022). Hao et al. (2024) analyzed the *SBP* family transcription factors in broomcorn millet and identified through real‐time fluorescence polymerase chain reaction that *PmSBP1* and *PmSBP7* were closely related to grain development and grain color in broomcorn millet. Li, Zhao, et al. (2021); Li, Wen, et al. (2021) demonstrated that nonanal, (Z)‐2‐nonenal, (E,E)‐2,4‐nonadienal, naphthalene dibutylphthalate, etc., are important compounds for distinguishing different color broomcorn millet varieties. Li et al. (2022) found that the selection of alleles related to yellow grains led to alterations in the metabolite profiles such as carotenoids and endogenous plant hormones, and identified metabolites with anti‐inflammatory properties (flavonoids, amides, and organic acids).

Shanxi is a genetic diversity center of broomcorn millet (Dong et al. 2015) and a main production area, with rich local and farmland germplasm resources (Wang et al. 2019). Research on broomcorn millet mainly focuses on its nutrition, flavor, antioxidant capacity, and health benefits (Liang and Liang 2019; Udeh et al. 2017; Devi et al. 2014; Li, Zhao, et al. 2021; Li, Wen, et al. 2021). However, there is little information on the compounds that provide these excellent properties in broomcorn millet. This study used five representative broomcorn millet varieties in Shanxi Province (YSNO13, YSNO16, JSNO9, JSNO15, and PSNO9) as materials and adopted the extensive targeted metabolomics method to analyze the types and relative contents of metabolites in the grains of different broomcorn millet varieties. This research provides an important theoretical basis for the genetic improvement and functional component development and utilization of broomcorn millet and has guiding significance for promoting the research and development of high‐value products of broomcorn millet.

## 2 Materials and Methods

### 2.1 Experimental Design

This study used different broomcorn millet varieties in Shanxi, including Yanshu No. 13 (YSNO13), Yanshu No. 16 (YSNO16), Jinshu No. 9 (JSNO9), Jinshu No. 15 (JSNO15), and Pingshu No. 9 (PSNO9) as experimental materials. After harvesting the unhusked whole grains, put them into polyethylene bags and store them in a refrigerator at −20°C.

### 2.2 Measurement Indicators and Methods

#### 2.2.1 Sample Preparation and Extraction for Widely Targeted Metabolomics Analysis

The sample preparation and extraction were carried out according to the method of Dunn et al. (2011). The sample was first ground (60 Hz, 60 s), 50 mg was weighed into a centrifuge tube, and 700 μL of the extract (meol‐water 3:1, pre‐cooled at −40°C, including internal standard) was added. After vortexing for 30 s, homogenize at 40 Hz for 4 min and ultrasonicate in an ice water bath for 5 min. This homogenization and ultrasonic step is repeated three times. Subsequently, it was placed overnight on a 4°C mixer. The next day, it was centrifuged at 12,000 RPM (centrifugal force 13,800 (×g), radius 8.6 cm) at 4°C for 15 min. The supernatant was then filtered through a 0.22 μm filter membrane. Dilute the supernatant with the extract 20 times, vortex for 30 s, and take 50 μL of each to mix as the QC sample. Store at −80°C until on‐machine testing.

#### 2.2.2 Instrument Parameters

According to the method of Chen et al. (2013), the target compound was chromatographically separated by using the EXION LC System (SCIEX) ultra‐performance liquid chromatograph through the Waters UPLC liquid chromatographic column. The mobile phase of the liquid chromatography was 0.1% formic acid water (A) and acetonitrile (B). The chromatographic column was kept at a constant temperature of 40°C, and the automatic sampler was at 4°C. The injection volume was 2 μL. Mass spectrometry detection was carried out using the SCIEX 6500 QTRAP+ system, IonDrive Turbo V ESI ion source, and multi‐reaction monitoring (MRM) scanning mode. Ion source parameters were as follows: IonSpray Voltage: +5500/−4500 V, Curtain Gas: 35 psi, Temperature: 400°C, Ion Source Gas 1: 60 psi, Ion Source Gas 2: 60 psi, DP: ±100 V.

### 2.3 Data Analysis

The Kyoto Encyclopedia of Genes and Genomes (KEGG) database (http://www.kegg.jp/kegg/pathway.html) was used for annotation and classification of differential metabolites. Pathways significantly enriched with metabolites in the module were compared with the background, and a threshold of *p* < 0.01 was used for definition. All data were processed using Excel (2022) and SPSS 27.0 (SPSS Institute Inc., Chicago, USA) software. Statistical analysis and mapping were performed using Origin (2024) and AI (2024) software. Analysis of variance and multiple comparisons were conducted using the LSD method (*p* < 0.05), and data for each indicator were expressed as the mean ± standard error of three replicates.

## 3 Results and Analysis

### 3.1 Quality Control of Metabolomics Data

**FIGURE 1:**
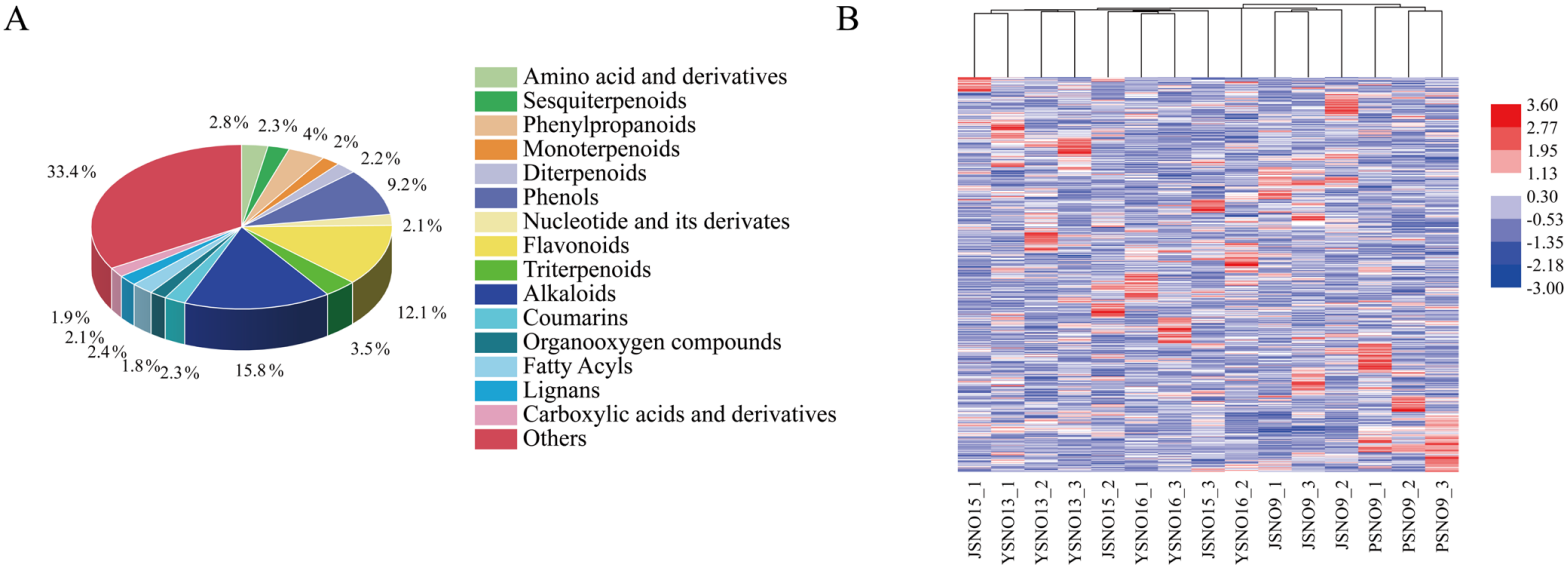
The diversity and variability of metabolites in different varieties of broomcorn millet. (A) Classification pie chart. (B) Cluster heat map analysis.

To gain insight into the distribution and variation of metabolites in different broomcorn millet varieties, we performed widely targeted metabolomics profiling on the grains of YSNO13, YSNO16, JSNO9, JSNO15, and PSNO9. Using the widely targeted metabolomics (LC–MS) method, we detected metabolites in five broomcorn millet varieties and identified a total of 1119 metabolites, which were classified into 16 major categories (Figure 1A). The main categories included phenols (108), alkaloids (185), amino acids and derivatives (33), sesquiterpenoids (27), phenylpropanoids (47), monoterpenoids (23), diterpenoids (26), nucleotides and their derivatives (25), flavonoids (142), triterpenoids (41), coumarins (27), organooxygen compounds (21), fatty acyls (28), lignans (24), carboxylic acids and derivatives (22), and others (391).

The hierarchical clustering heatmap constructed based on standardized metabolite content (Figure 1B) shows that the samples can be clearly clustered into five categories, indicating a significant correlation between metabolic profiles and varieties. The PCA analysis results (Figure S1) show that the cumulative contribution rate of the first two principal components (PC1 = 7.9%, PC2 = 5.3%) reaches 13.2%. The sample points of the five varieties show obvious separation, with good experimental repeatability and reliable data. This result confirmed that the five varieties of broomcorn millet have significantly different metabolic characteristic profiles. The extraction ion chromatogram of the sample is detailed in attached Figure S1. In the figure, the chromatographic peaks of all target compounds present symmetrical shapes, indicating that good chromatographic separation has been achieved for each target compound. The Pearson coefficient of the QC sample (Figure S2) indicates that the detection process has good repeatability and the instrument stability is also good, which provides a guarantee for the accuracy of the experimental data. To confirm the observed changes in metabolite profiles, we performed OPLS‐DA analysis (Figure S3). In all pairwise comparisons, the value of R^2^Y was greater than that of Q^2^Y, which indicates that the repeatability and stability of the samples detected in this study are better, the model is reliable, and it can be applied to subsequent metabolic analysis processes such as differential metabolite screening.

### 3.2 Screening and Enrichment Analysis of Differential Metabolites (DAMs) in Different Broomcorn Millet Varieties

#### 3.2.1 Screening of DAMs in Different Broomcorn Millet Varieties

**FIGURE 2:**
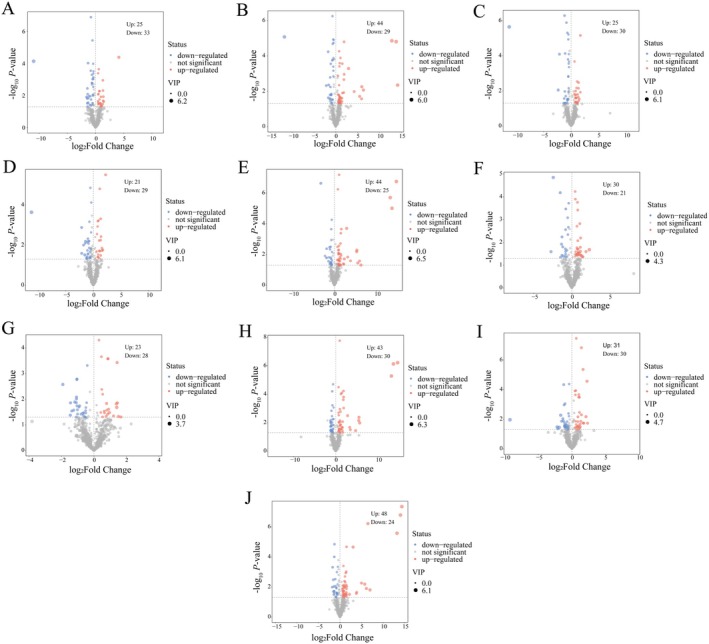
DAMs analysis of different broomcorn millet varieties. (A–J) DAMs volcano maps of JSNO9 versus JSNO15, JSNO9 versus PSNO9, JSNO9 versus YSNO13, JSNO9 versus YSNO16, JSNO15 versus PSNO9, JSNO15 versus YSNO13, JSNO15 versus YSNO16, YSNO13 versus PSNO9, YSNO13 versus YSNO16, and YSNO16 versus PSNO9.

To identify the most important metabolites in different broomcorn millet varieties, this study combined the OPLS‐DA model to calculate two parameters, the variable importance of projection (VIP) and *p* value. Metabolites were screened as differential metabolites (DAMs) with thresholds of VIP > 1.0, |log_2_FC| > 1.5 or < 0.667, and *p* < 0.05 (Figure 2). A total of 58 DAMs were screened out between JSNO9 versus JSNO15 (25 up‐regulated and 33 down‐regulated) (Table S1). Among JSNO9 versus PSNO9, 73 DAMs were screened out (44 were up‐regulated and 29 were down‐regulated) (Table S2). There were 55 DAMs screened out between JSNO9 versus YSNO13 (25 up‐regulated and 30 down‐regulated) (Table S3). Fifty DAMs were screened out between JSNO9 versus YSNO16 (21 up‐regulated and 29 down‐regulated) (Table S4). Among JSNO15 versus PSNO9, 69 DAMs were screened out (44 were up‐regulated and 25 were down‐regulated) (Table S5). Fifty‐one DAMs were screened out between JSNO15 versus YSNO13 (30 up‐regulated and 21 down‐regulated) (Table S6). A total of 51 DAMs were selected between JSNO15 versus YSNO16 (23 up‐regulated and 28 down‐regulated) (Table S7). A total of 73 DAMs were screened out between YSNO13 versus PSNO9 (43 up‐regulated and 30 down‐regulated) (Table S8). A total of 61 DAMs (31 up‐regulated and 30 down‐regulated) were screened out between YSNO13 and YSNO16 (Table S9). Among YSNO16 versus PSNO9, 72 DAMs were screened out (48 up‐regulated and 24 down‐regulated) (Table S10).

**FIGURE 3:**
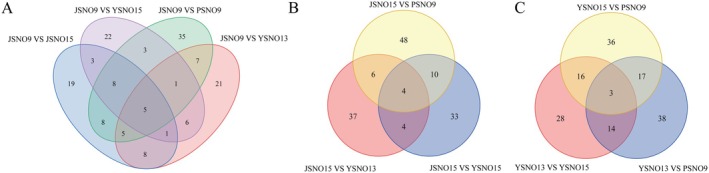
Venn diagram of different broomcorn millet varieties DAMs.

The Venn diagram indicates the DAMs relationship among different grain colors of broomcorn millet (Figure 3). Five common DAMs were found in the four groups of PSNO9 versus JSNO15, JSNO9 versus PSNO9, JSNO9 versus YSNO13, and JSNO9 versus YSNO16 (Figure 3A), namely glycerophosphocholine, palmitic acid, indole‐3‐acetic acid, (‐)‐gallocatechin, and n6‐isopentenyladenosine. Four common DAMs were found in the three groups of JSNO15 versus PSNO9, JSNO15 versus YSNO13, and JSNO15 versus YSNO16 (Figure 3B), namely beta‐carotene, n6‐isopentenyladenosine, gibberellin, and 2′‐o‐methyladenosine. Three common DAMs were found in the three groups of YSNO13 versus PSNO9, YSNO13 versus YSNO16, and YSNO16 versus PSNO9 (Figure 3C), namely indole‐3‐carboxaldehyde, cinnamyl, and calystegine.

#### 3.2.2 Enrichment Analysis of DAMs in Different Broomcorn Millet Varieties

**FIGURE 4:**
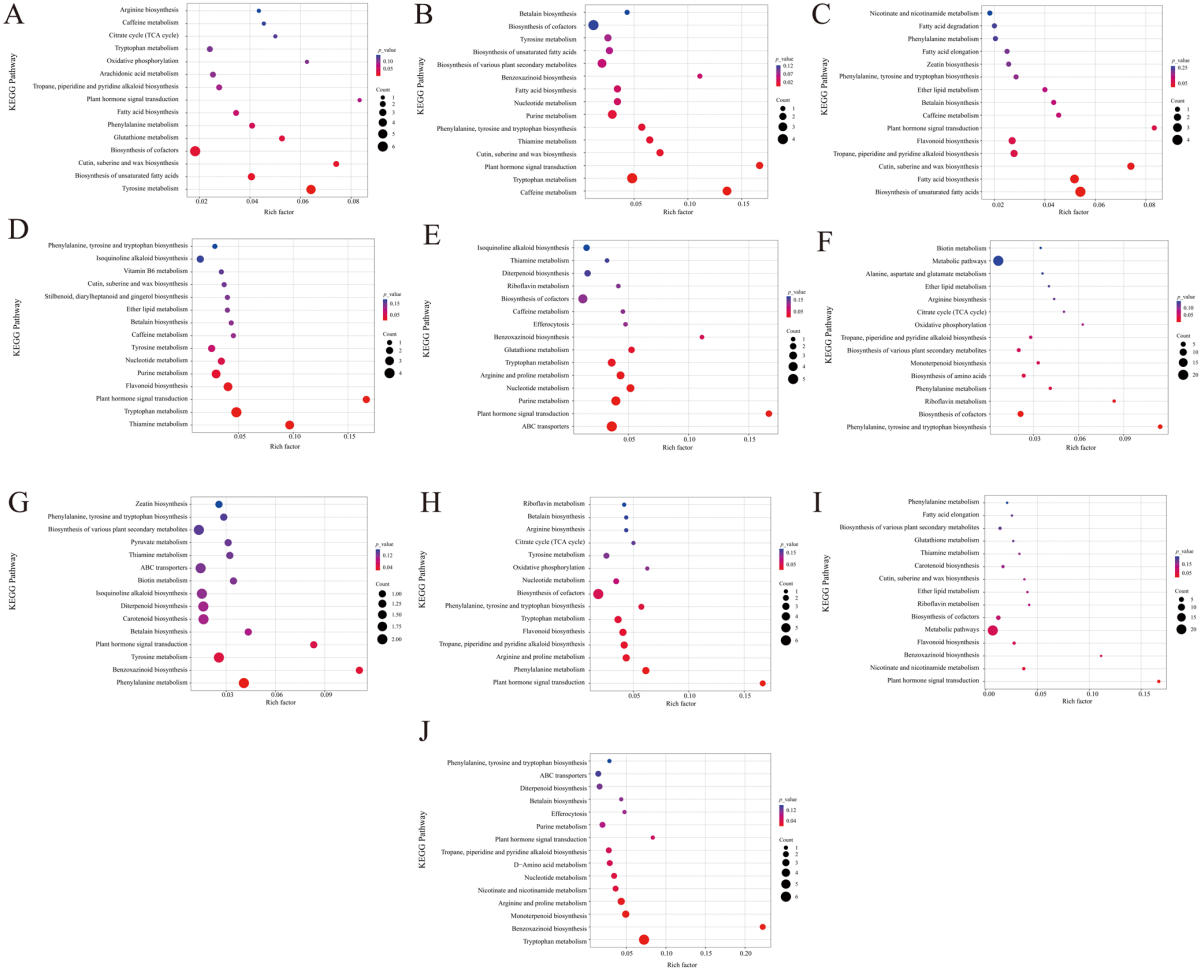
Enrichment maps of KEGG pathways in five varieties of broomcorn millet. (A–J) Enrichment maps of JSNO9 versus JSNO15, JSNO9 versus PSNO9, JSNO9 versus YSNO13, JSNO9 versus YSNO16, JSNO15 versus PSNO9, JSNO15 versus YSNO13, JSNO15 versus YSNO16, YSNO13 versus PSNO9, YSNO13 versus YSNO16, YSNO16 versus PSNO9. The vertical axis in the figure represents the metabolic pathway, and its horizontal axis, along with the size of the bubble, jointly indicate the magnitude of the influencing factors of this pathway. The larger the bubble, the greater the influencing factor. The bubble color represents the *p*‐value of the enrichment analysis. The darker the color, the higher the enrichment degree.

KEGG pathway enrichment analysis was performed to further determine the significantly (*p* < 0.01) enriched metabolic pathways among different broomcorn millet varieties (Figure 4). The DAMs between JSNO9 versus JSNO15 were mainly enriched in the tyrosine metabolism pathway. The DAMs of JSNO9 versus YSNO13 were mainly enriched in the biosynthesis of unsaturated fatty acids, fatty acid biosynthesis, and cutin, suberine, and wax biosynthesis pathways. The DAMs between JSNO9 versus YSNO16 were mainly enriched in tryptophan metabolism, plant hormone signal transduction, and thiamine metabolism pathways. The DAMs between JSNO9 versus PSNO9 were mainly enriched in tryptophan metabolism, caffeine metabolism, cutin, suberine, and wax biosynthesis, and plant hormone signal transduction pathways. The DAMs between JSNO15 versus YSNO13 were mainly enriched in the riboflavin metabolism pathway. The DAMs between JSNO15 versus PSNO9 were mainly enriched in abc transporters, purine metabolism, nucleotide metabolism, and arginine and proline metabolism pathways. The DAMs between YSNO13 versus YSNO16 were mainly enriched in the plant hormone signal transduction pathway. The DAMs between YSNO13 versus PSNO9 were mainly enriched in phenylalanine metabolism and plant hormone signal transduction pathways. The DAMs between YSNO16 versus PSNO9 were mainly enriched in nucleotide metabolism, d‐amino acid metabolism, and tropane, piperidine, and pyridine alkaloid biosynthesis pathways.

### 3.3 Changes in Phenolic Compounds in Different Broomcorn Millet Varieties

#### 3.3.1 Phenolic Acids

**FIGURE 5:**
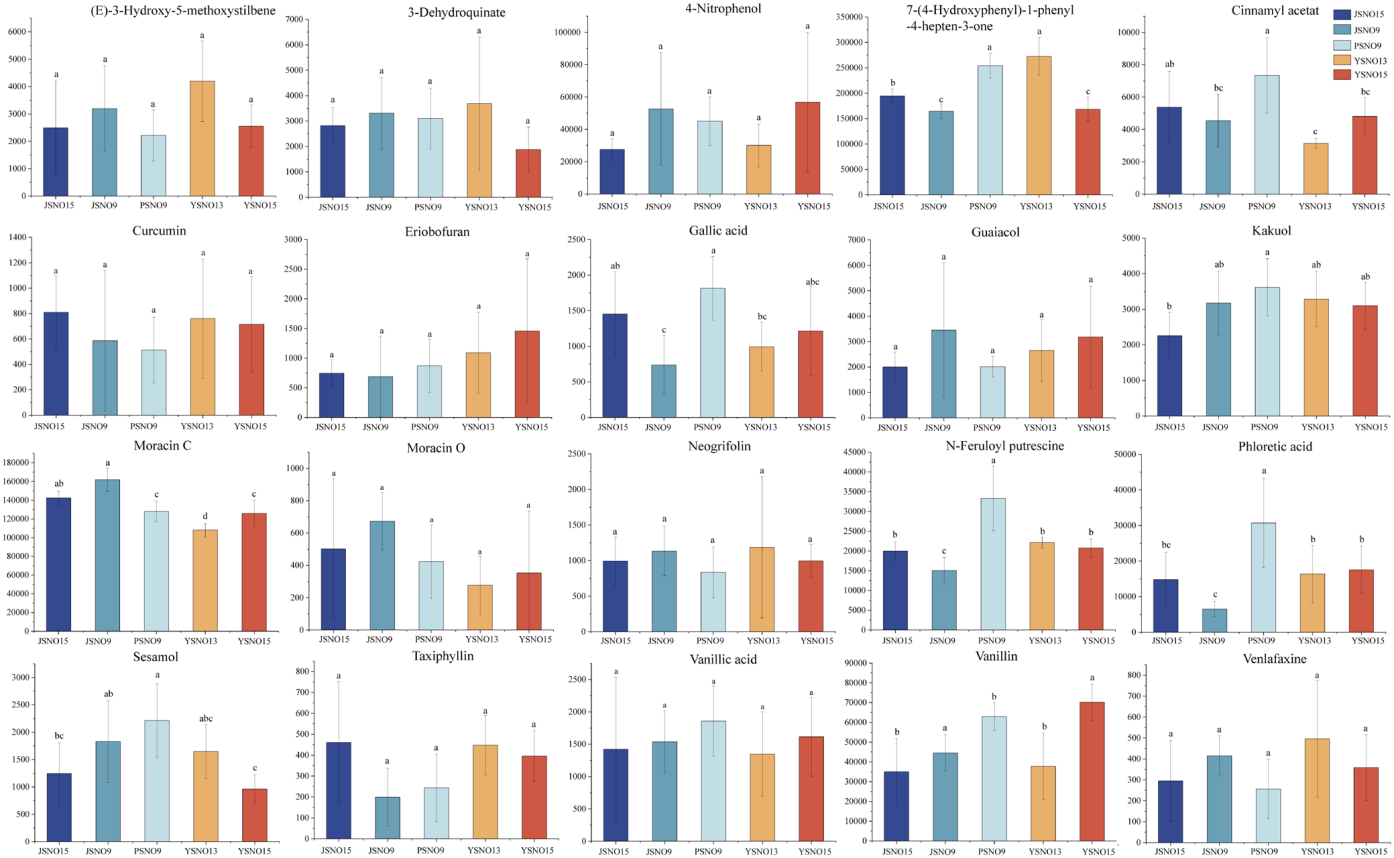
Peak areas of 20 kinds of phenolic acids identified in different varieties of erosion. Different letters indicate significant differences (*p* < 0.05).

A total of 20 phenolic acids were detected in broomcorn millet with different grain colors (Figure 5), including four hydroxybenzoic acid derivatives (gallic acid, vanillic acid, phloretic acid, and ethylparaben) and two hydroxycinnamic acid derivatives (n‐feruloyl putrescine and cinnamyl acetate). 7‐(4‐Hydroxyphenyl)‐1‐phenyl‐4‐hepten‐3‐one had the highest content in YSNO13. Cinnamyl acetate, kakuol, gallic acid, and sesamol had the highest contents in PSNO9. Moracin C had the highest content in JSNO9. Vanillin had the highest content in YSNO16. Cinnamyl acetate had the lowest content in YSNO13. Gallic acid had the lowest content in JSNO9. Sesamol had the lowest content in YSNO16. Phloretic acid had the lowest content in JSNO9.

#### 3.3.2 Flavonoids

**FIGURE 6:**
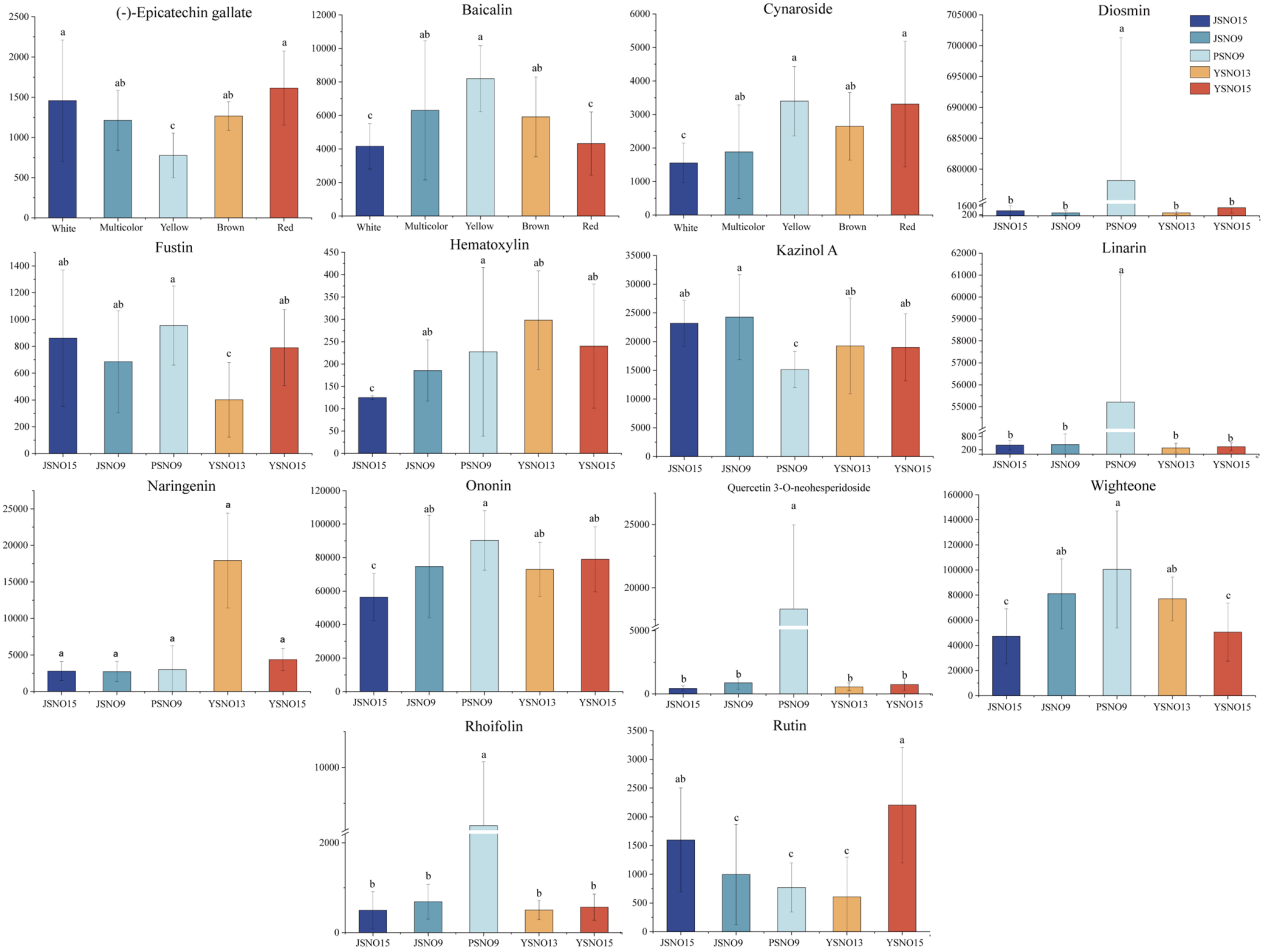
Peak areas of a total of 14 flavonoids identified in different varieties of broomcorn millet. Different letters indicated significant differences (*p* < 0.05).

Fourteen flavonoid compounds with significant differences were screened from different broomcorn millet varieties (Figure 6), including six flavonoids (wighteone, linarin, kazinol a, quercetin 3‐o‐neohesperidoside, hematoxylin, fus tin) and eight flavonoids (naringenin, (‐)‐epicatechin gallate, baicalin, diosmin, rutin, ononin, cynaroside, and rhoifolin). Baicalin, cynaroside, fustin, hematoxylin, diosmin, linarin, quercetin 3‐o‐neohesperidoside, wighteone, rhoifolin, and ononin had the highest contents in PSNO9. (‐)‐Epicatechin gallate and rutin had the highest contents in YSNO16. Kazinol A had the highest content in JSNO9. Naringenin had the highest content in YSNO13.

#### 3.3.3 Amino Acids and Their Derivatives

**FIGURE 7:**
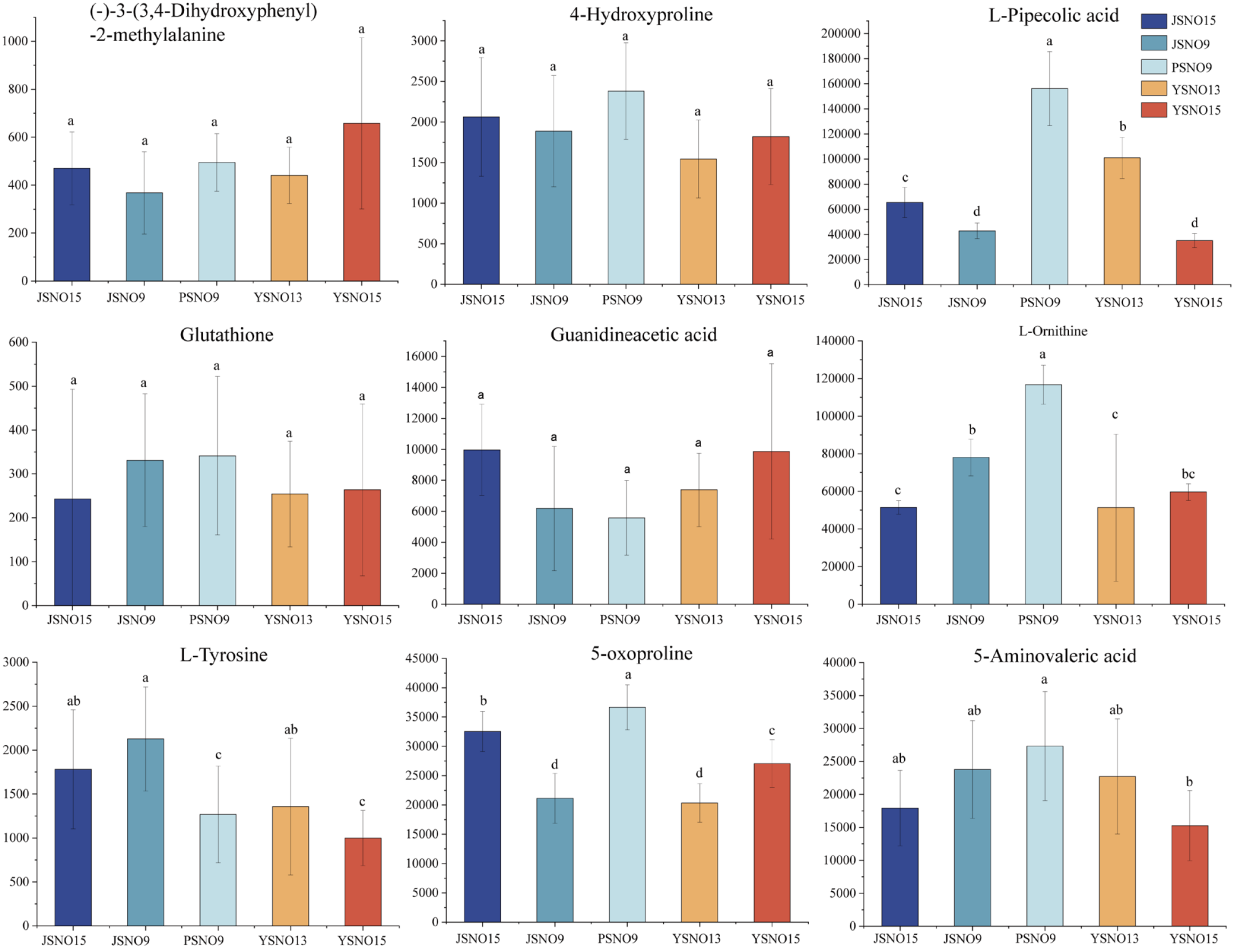
Peak areas of a total of 9 amino acids and their derivatives identified in different varieties of broomcorn millet. Different letters indicate significant differences (*p* < 0.05).

Nine amino acids and their derivatives were screened from broomcorn millet with different grain colors (Figure 7). 5‐Aminovaleric acid, l‐ornithine, L‐pipecolic acid, and 5‐oxoproline had the highest contents in PSNO9. L‐Tyrosine had the highest content in JSNO9. (‐)‐3‐(3,4‐Dihydroxyphenyl)‐2‐methylalanine, 4‐hydroxyproline, guanidineacetic acid, and glutathione showed no significant differences among the five broomcorn millet varieties.

### 3.4 Analysis of Key Metabolic Pathways in Different Broomcorn Millet Varieties

**FIGURE 8:**
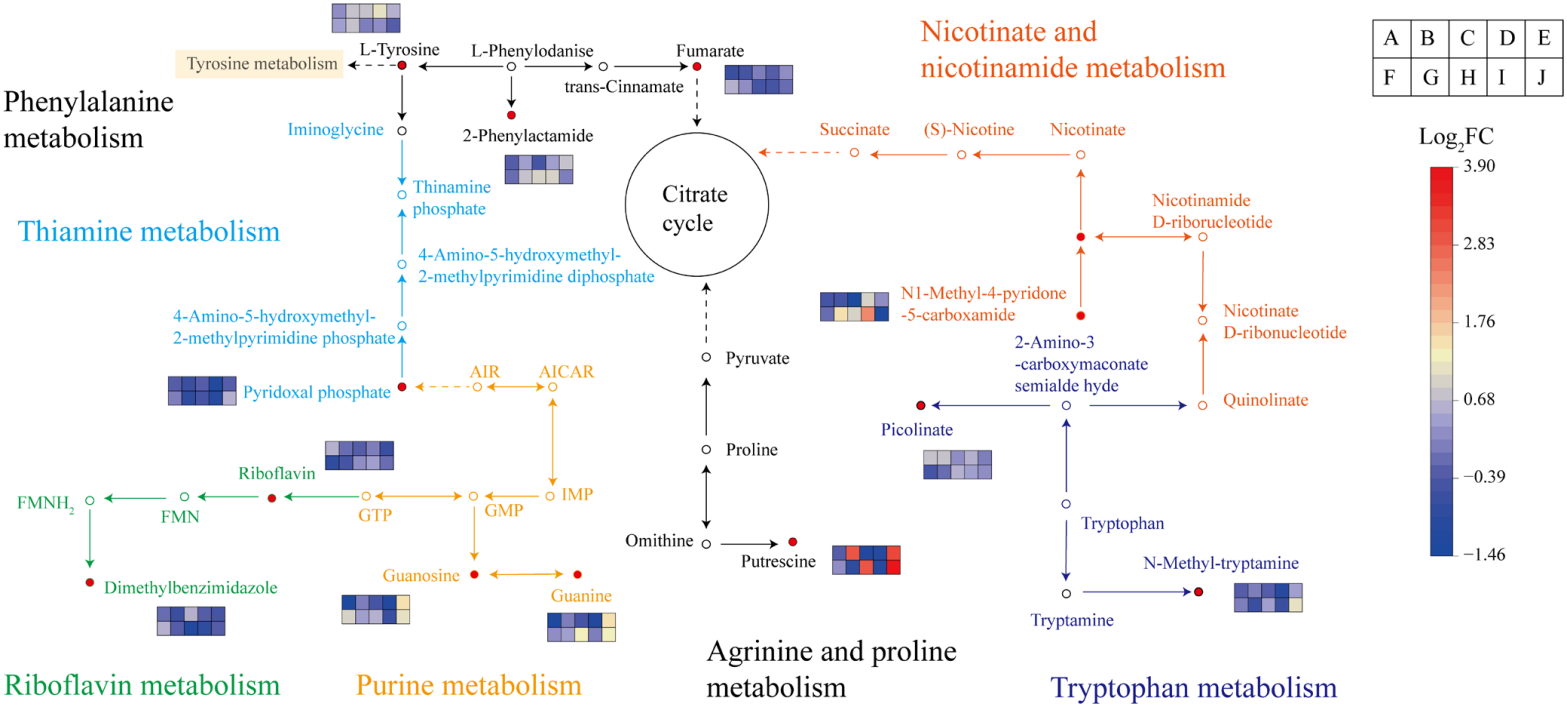
Analysis of significant enrichment pathways of different broomcorn millet varieties. Rectangles A and J respectively represent the log_2_FC of key metabolites between each comparison group (JSNO9 versus JSNO15, JSNO9 versus PSNO9, JSNO9 versus YSNO13, JSNO9 versus YSNO15, JSNO15 versus PSNO9, JSNO15 versus YSNO13, JSNO15 versus YSNO15, YSNO13 versus PSNO9, YSNO13 versus YSNO15, and YSNO15 versus PSNO9). The red circles represent the differential metabolites whose contents change, while the white circles represent the metabolites whose contents remain unchanged.

Further analysis was conducted on the pathways significantly enriched (*p* < 0.01) in different varieties of broomcorn millet (Figure 8). The DAMs between JSNO9 versus JSNO15 were significantly enriched in the phenylalanine metabolism pathway, in which L‐tyrosine was significantly up‐regulated, and fumaric acid and 2‐phenylacetamide were significantly down‐regulated. The DAMs between JSNO9 versus YSNO13 were significantly enriched in tryptophan metabolism, purine metabolism, and thiamine metabolism pathways, with n‐methyltryptamine significantly down‐regulated, picolinic acid significantly up‐regulated, guanosine and L‐tyrosine significantly up‐regulated, and pyridoxal phosphate significantly down‐regulated. The DAMs between JSNO9 versus PSNO9 were significantly enriched in purine metabolism and thiamine metabolism pathways, with guanine significantly down‐regulated and L‐tyrosine significantly up‐regulated. The DAMs between JSNO15 versus YSNO13 were significantly enriched in the riboflavin metabolism pathway, with riboflavin significantly down‐regulated and dimethylbenzimidazole significantly up‐regulated. The DAMs between JSNO15 versus YSNO15 were significantly enriched in the phenylalanine metabolism pathway, with L‐tyrosine and 2‐phenylacetamide significantly up‐regulated. The DAMs between JSNO15 versus PSNO9 were significantly enriched in the purine metabolism pathway, with guanine and guanosine significantly up‐regulated. The DAMs between YSNO13 versus YSNO15 were significantly enriched in the nicotinate and nicotinamide metabolism pathway, with n1‐methyl‐4‐pyridone‐3‐carboxamide significantly up‐regulated. The DAMs between YSNO13 versus PSNO9 were significantly enriched in the phenylalanine metabolism pathway, with fumaric acid significantly down‐regulated and 2‐phenylacetamide significantly up‐regulated. The DAMs between YSNO15 versus PSNO9 were significantly enriched in the arginine and proline metabolism pathway, with putrescine significantly up‐regulated.

## 4 Discussion

### 4.1 Identification of DAMs in Different Broomcorn Millet Varieties

The natural gluten‐free property of broomcorn millet has solved the dietary restriction problem for patients with celiac disease (Das et al. 2019). It contains richer proteins, vitamins, and various minerals (Li, Zhao, et al. 2021; Li, Wen, et al. 2021). These characteristics make its application value in food and healthy diets increasingly prominent. Although broomcorn millet possesses these excellent qualities, due to the insufficient development and utilization of its nutritional value and functional active components, there are still certain limitations in its application fields at present. In this study, a total of 1119 compounds were identified based on the results of metabolomics. We identified a total of 20 phenolic acids, 14 flavonoids with significant differences, and 9 amino acids and their derivatives. Among them, the contents of 5 phenolic compounds were significantly higher, including phloretic acid, n‐feruloyl putrescine, moracin c, 7‐(4‐Hydroxyphenyl)‐1‐phenyl‐4‐hepten‐3‐one, and 4‐nitrophenol, etc. The contents of 4 flavonoid compounds were significantly higher, including wighteone, kazinol a, ononin, and dihydromyricetin, etc. The contents of 4 amino acids and their derivatives were significantly higher, including L‐pipecolic acid, guanidineacetic acid, L‐ornithine, 5‐aminovaleric acid, and 5‐oxoproline. N‐Feruloyl putrescine has been shown to have antioxidant (Choi et al. 2007) and anti‐inflammatory (Kim et al. 2012) effects. In addition, 185 kinds of alkaloids, 27 kinds of sesquiterpenoids, and 25 kinds of nucleotides and their derivatives and other metabolites were identified. A total of 1119 metabolites identified in five different types of broomcorn millet grains were compared and verified with the previously identified secondary metabolites, providing reference value for the isolation and identification of functional components of broomcorn millet.

**FIGURE 9:**
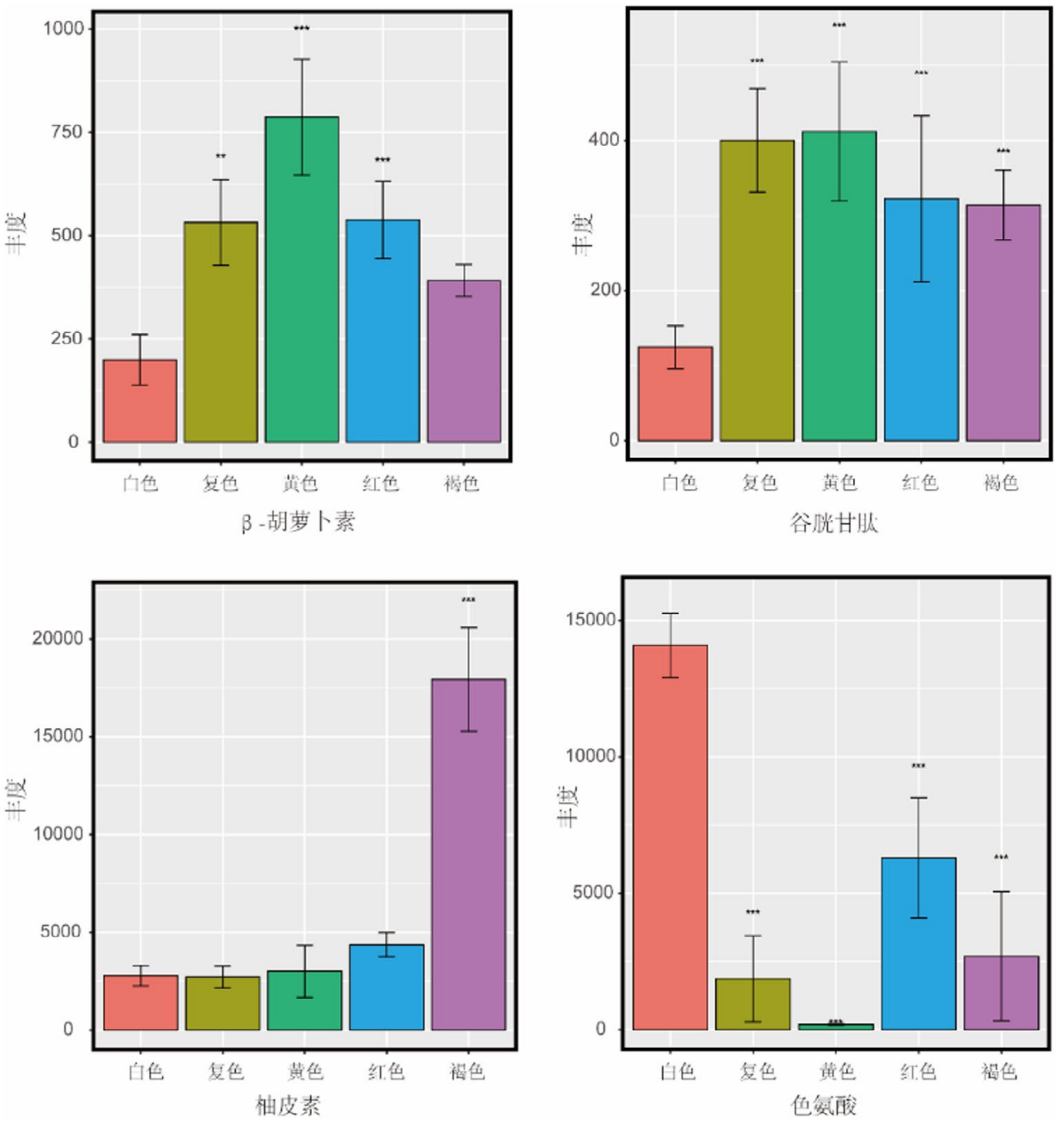
Distribution of abundance of color‐related metabolites in different broomcorn millet varieties.

As an important vitamin and natural pigment, β‐carotene is the metabolite that is mainly focused on in this study. He et al. (2022) found that carotenoid cleavage dioxygenase 1 catalyzes lutein degradation to influence carotenoid accumulation and color development in broomcorn millet grains. The carotenoid content in grains can be estimated by measuring the yellow color of grains using a colorimeter (Yano et al. 2016). We found that the abundance of terpenoids was related to the grain color of broomcorn millet (Figure 9). In this study, the abundance of β‐carotene in colored grains was 196% to 395% of that in white grains (Figure 9). β‐Carotene belongs to tetraterpenoids, naturally presenting orange‐yellow or orange‐red, and is one of the main pigments affecting pericarp color in many fruits (Dumont et al. 2020). These data suggest that β‐carotene is also related to the grain color of broomcorn millet.

Matsui et al. (2018) found that the accumulation level of glutathione was significantly correlated with the color of broomcorn millet grains and might affect the deposition of plant pigments by regulating the biosynthesis pathway of anthocyanins. A genome‐wide association study showed that glutathione metabolism genes are candidate genes for the QTL of broomcorn millet inflorescence color (Chen et al. 2023). This study found that PSNO9 had the highest glutathione content, followed by JSNO9. Zhao et al. (2022) found that red adzuki beans have more than ten kinds of skin colors, which are closely related to the metabolic pathways of anthocyanins and flavonoids. Naringenin is an important intermediate product required for anthocyanin synthesis in the anthocyanin synthesis pathway (Holton and Cornish 1995). The metabolome data of this study show that the abundance of glutathione and naringenin in colored grains is higher than that of JSNO15. The abundance of glutathione and naringenin in YSNO13 was 2.9 and 4.1 times that of JSNO15, respectively (Figure 9). These metabolite data indicate that the anthocyanin synthesis pathway is related to the grain color of broomcorn millet.

Amino acid metabolism is also related to the color of broomcorn millet grains, among which Tryptophan is included (Wang et al. 2024). Tryptophan is one of the essential amino acids for the human body and plays a significant role in maintaining intestinal health and immune function (Wang et al. 2024). This study found that the DAMs between JSNO9 versus YSNO16 and JSNO9 versus PSNO9 were commonly enriched in the tryptophan metabolism pathway. A genome‐wide association study showed that tryptophan decarboxylase is a candidate gene for the QTL of broomcorn millet grain color (Chen et al. 2023), which can convert L‐tryptophan into tryptamine. The higher the abundance of tryptamine, the darker the grain color (Kanjanaphachoat et al. 2012). Similarly, the lower the abundance of L‐tryptophan in the grains, the darker the color. Correspondingly, in the metabolome data of this study, the abundance of L‐tryptophan in colored grains was lower than that in JSNO15. The abundance of L‐tryptophan in JSNO15 is 1.78 times that of YSNO13 (Figure 9). Li et al. found that after the differentially expressed genes were annotated in the KEGG database, it was revealed that tryptophan metabolism and metabolic pathways were both associated with the color of broomcorn millet grains (Li et al. 2023). This study found that the DAMs between JSNO9 versus YSNO16 and JSNO9 versus PSNO9 were commonly enriched in the tryptophan metabolism pathway. The 1119 metabolites identified in 5 kinds of broomcorn millet grains were compared with the previously identified secondary metabolites, which provided a reference value for the separation and identification of functional components in broomcorn millet.

### 4.2 Identification of Phenolic Compounds Among Different Broomcorn Millet Varieties

In this study, 562 DAMs were identified in 10 comparison groups. The DAMs screened between JSNO9 versus JSNO15, JSNO9 versus PSNO9, JSNO9 versus YSNO13, JSNO9 versus YSNO16, JSNO15 versus PSNO9, JSNO15 versus YSNO13, JSNO15 versus YSNO16, YSNO13 versus PSNO9, YSNO13 versus YSNO16, and YSNO16 versus PSNO9 were 58, 73, 55, 50, 69, 51, 51, 73, 61, and 72 species, respectively. Li, Zhao, et al. (2021); Li, Wen, et al. (2021) compared the main pathways of white, black, gray, and red broomcorn millet, including tryptophan metabolism, flavonoids, isoflavones, flavones, and flavonol biosynthesis. This study found that DAMs between JSNO9 versus YSNO16 and JSNO9 versus PSNO9 were mainly enriched in tryptophan metabolism. Only the tyrosine metabolism pathway of JSNO9 versus JSNO15 had an enrichment analysis with *p* < 0.01, indicating significant differences in tyrosine metabolism between JSNO9 versus JSNO15. PSNO9 had the highest contents of flavonoid compounds (10), phenolic compounds (9), and amino acids and their derivatives (6). It has been reported that flavonoid compounds have health benefits such as anti‐cancer, anti‐microbial, antioxidant, anti‐inflammatory, anti‐fungal, anti‐ulcer, and anti‐edema activities (Ekalu and Habila 2020).

Phenolic compounds have significant antibacterial activity (Luo and Fang 2023). They can inhibit the growth of bacteria by interfering with the synthesis of their cell walls and DNA replication (Luo and Fang 2023). They effectively maintain physical health by regulating the level of free radicals in the body, inhibiting inflammatory responses, and enhancing immune function (Park et al. 2018), removing reactive oxygen species and chelating metal ions at the active sites of metalloenzymes, reducing excessive pigment deposition, and exerting whitening effects (Kim et al. 2004; Fan et al. 2017). Amino acids and their derivatives have extensive nutritional value and functional effects. There is a significant correlation between the color of quinoa grains and their amino acid profile (Qian et al. 2021). The research results of Qian et al. (2021) suggest that the color differences of quinoa grains of different colors may be closely related to their amino acid metabolic pathways. Broomcorn millet contains more essential amino acids than other gluten‐free grains (Wiedemair et al. 2020). Zhang et al. (2023) found that there were significant differences in the composition of amino acids and their derivatives in waxy grains and mature grains between the two types of wheat. The results of this study showed that 5‐aminovaleric acid, L‐ornithine, L‐pipecolic acid, 5‐oxoproline, and L‐tyrosine were significantly different among different broomcorn millet varieties (Figure 8). JSNO9 had the highest L‐tyrosine content, which was 19.38% higher than that of JSNO15. Moreover, L‐tyrosine was significantly down‐regulated between JSNO15 versus PSNO9, JSNO15 versus YSNO13, and JSNO15 versus YSNO15 (Figure 9). L‐Tyrosine can be used in nutritional supplements and food additives, etc. (Deng et al. 2023; Wang et al. 2023).

Studies have shown that the grain color of broomcorn millet is related to phenolic substances in the seed coat. Ma et al. (2016) found that there were significant differences in the content of phenolic acids in wheat grains of different colors. Ferulic acid dominates in red and yellow wheat, vanillic acid is in blue wheat, and para‐vanillic acid is in purple wheat (Paznocht et al. 2020). The darker the grain color, the higher the content and more types of phenolic acids. Pradeep and Sreerama (2015) analyzed the content and type of phenolic acids in raw and processed broomcorn millet and identified 8 phenolic acids, among which vanillic acid was the main phenolic acid detected. In this study, vanillic acid had the highest content in PSNO9, which was 30.70% higher than that in JSNO15 (Figure 5). Vanillic acid can improve blood sugar levels and enhance antioxidant activity (Vinothiya and Ashokkumar 2017). It also has anti‐cancer (Gong et al. 2019) and anti‐fungal (Khan et al. 2024) properties, highlighting its wide biomedical applications. In addition, vanillic acid helps with wound healing, promotes cell regeneration, and protects multiple tissues from oxidative stress (Zhu et al. 2024). In this study, there were significant differences in the relative contents of phenolic acid substances such as cinnamyl acetate, kakuol, gallic acid, and sesamol in different colored broomcorn millet grains (Figure 6). The differences in the antioxidant activity of broomcorn millet may be related to the varying contents of phenolic acids (Li, Zhao, et al. 2021; Li, Wen, et al. 2021).

Flavonoids are important secondary metabolites synthesized by the branch pathway of the phenylalanine metabolism pathway and belong to polyphenols (Naing et al. 2018). Chen et al. (2019) found that there are differences in the flavonoid biosynthesis pathways among different colored rice varieties, which reflect their differences in physiological functions. PSNO9 had the highest contents of flavonoid compounds such as baicalin, cynaroside, fustin, hematoxylin, diosmin, linarin, quercetin 3‐o‐neohesperidoside, wighteone, rhoifolin, and ononin. In this study, baicalin had the highest content in PSNO9, which was 96.82% higher than that in JSNO15 (Figure 7). Baicalin has a very significant antioxidant capacity. It can eliminate free radicals and inhibit lipid peroxidation reactions at the same time, thereby protecting cells from damage caused by oxidative stress (Biswas et al. 2010). PSNO9 has the highest cynaroside content, followed by YSNO16 (Figure 6). Cynaroside has significant antioxidant capacity, which can eliminate free radicals and reduce the production of reactive oxygen species, thereby protecting cells from oxidative stress damage and helping to improve the stress resistance of crops (Bouyahya et al. 2023). It is indicated that PSNO9 and YSNO16 have stronger stress resistance compared to other varieties. The data of this study show that the abundance of various compounds with antioxidant effects in colored broomcorn millet is higher than that in JSNO15. The identification of these metabolites with differences will be helpful for evaluating the functions and nutrition of different varieties of broomcorn millet.

## 5 Conclusion

This study used a widely targeted metabolomics approach to systematically analyze the grain metabolites of different broomcorn millet varieties. Metabolomics data revealed metabolic differences and functional material bases among varieties. A total of 1119 metabolites were identified in this study, which were classified into 16 major categories such as phenols, alkaloids, and phenylpropanoids. Among these metabolites, there are significant differences in the accumulation of phenolic acids, flavonoids, and amino acids, which are the key substances causing the differentiation of metabolic characteristics among different varieties. Differential metabolite analysis showed that metabolic differences among varieties were mainly enriched in pathways such as tryptophan metabolism, purine metabolism, and phenylalanine metabolism, and grain color was closely related to anthocyanin synthesis and tryptophan metabolism pathways. This study extensively targeted metabolomics to analyze the material basis of the variety differences of broomcorn millet and clarified the key roles of phenolic, flavonoid, and amino acid compounds in variety differentiation. It provides theoretical support for the genetic breeding, quality improvement, and functional component development of broomcorn millet, which is of great significance for promoting the efficient utilization of millet resources.

## Author Contributions

**Chao Jiang:** conceptualization (equal), data curation (lead), formal analysis (lead), methodology (equal), project administration (lead), resources (lead), supervision (lead), visualization (lead), writing – original draft (lead). **Jiao Mao:** conceptualization (equal), investigation (equal), methodology (equal), writing – review and editing (equal). **Xiaoqiang Song:** conceptualization (equal), supervision (equal), writing – review and editing (equal). **Hai Lia:** conceptualization (equal), supervision (equal), writing – review and editing (equal). **Xiaoning Cao:** methodology (equal), visualization (equal), writing – original draft (supporting), funding acquisition.

## Ethics Statement

The authors have nothing to report.

## Conflicts of Interest

The authors declare no conflicts of interest.

## Supporting information


**Data S1:** Supporting Information.

## Data Availability

The data that support the findings of this study are available on request from the corresponding author. The data are not publicly available due to privacy or ethical restrictions.
