# Correction to “Metabolomics Revealed the Differential Metabolites of Different Broomcorn Millet Varieties in Shanxi”

**DOI:** 10.1002/fsn3.71036

**Published:** 2025-09-26

Jiang, C., J. Mao, X. Song, H. Li, and X. Cao. 2025. “Metabolomics Revealed the Differential Metabolites of Different Broomcorn Millet Varieties in Shanxi.” *Food Science & Nutrition* 13, no. 9: e70902. https://doi.org/10.1002/fsn3.70902.

In “4.1 Identification of DAMs in Different Broomcorn Millet Varieties” of the “Discussion” section, Figure 9 is incorrect. We erroneously used the original version during the revision and proofreading of the article, resulting in the error. The correct figure appears below.

We apologize for this error.

**FIGURE 9:**
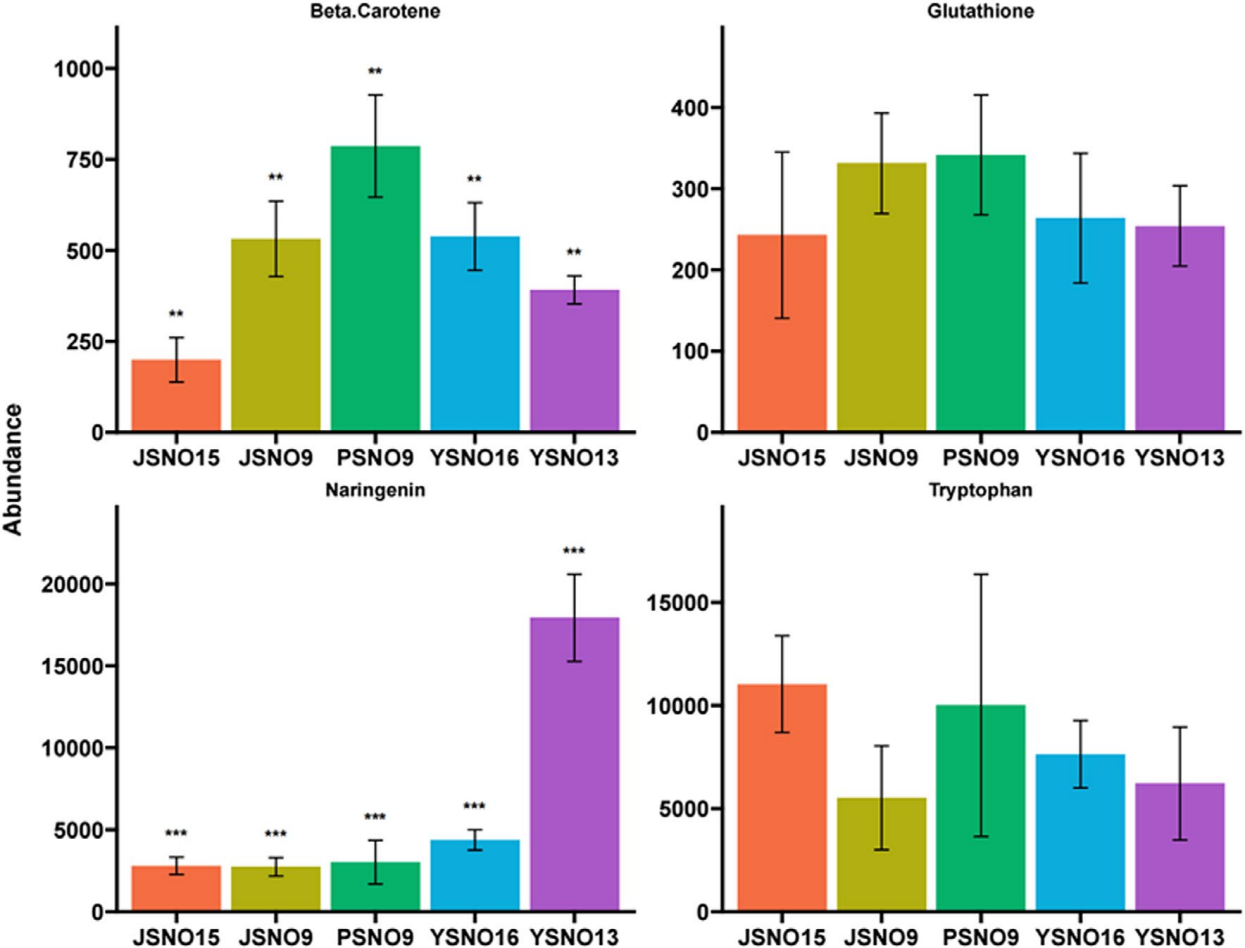
Distribution of abundance of color‐related metabolites in different erosive varieties.

