# Supplementary material for: Metabolomics Revealed the Differential Metabolites of Different Broomcorn Millet Varieties in Shanxi
Source: Food Sci Nutr. 2025 Sep 3;13(9):e70902. doi: 10.1002/fsn3.70902 (PMC12406176; doi:10.1002/fsn3.70902)
Supplement: Supplementary file 1 — Data S1: Supporting Information. [file FSN3-13-e70902-s001.docx]

Supplementary material

**
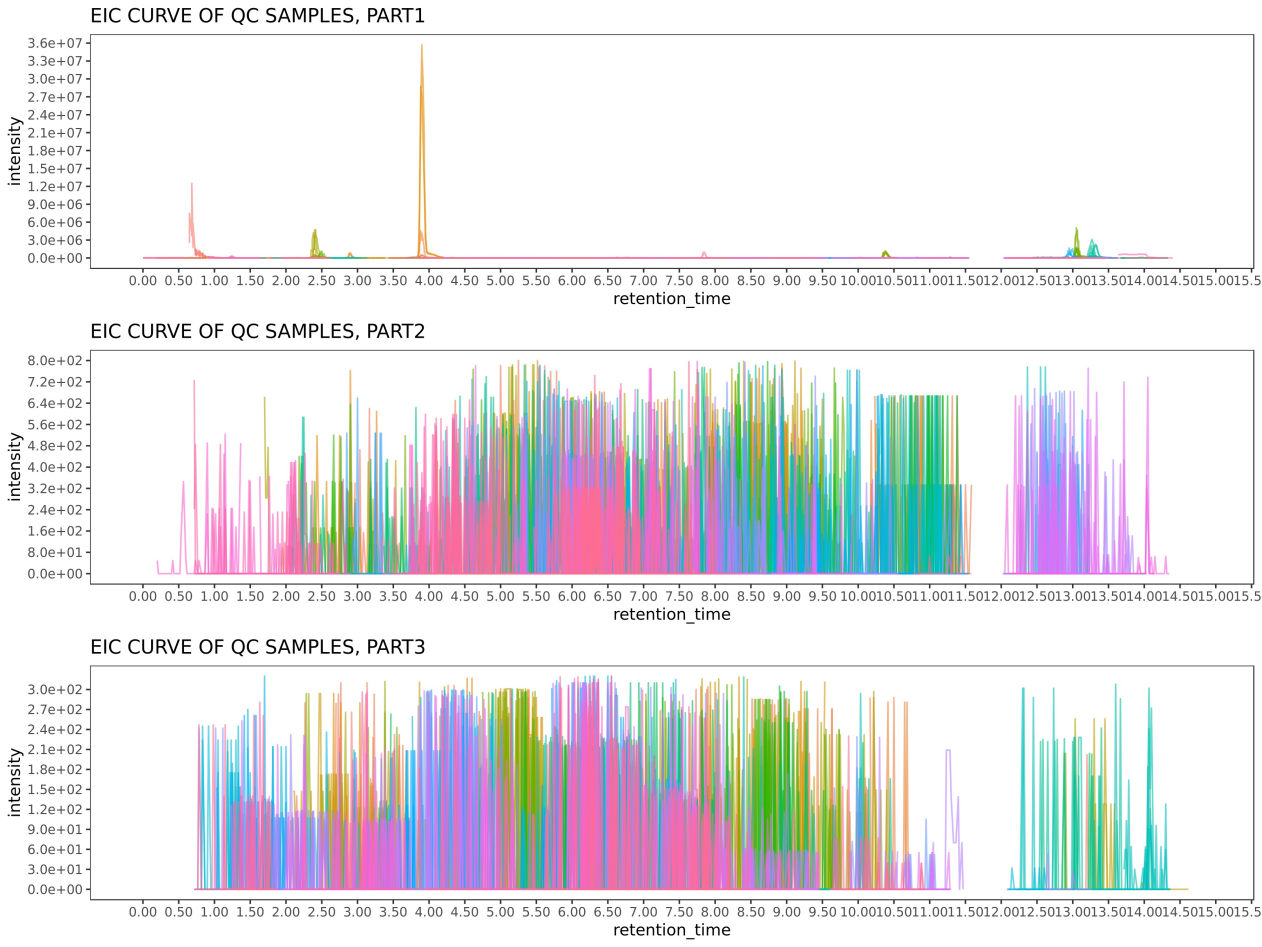
**

Fig. S1. Ion current diagram of QC sample extraction


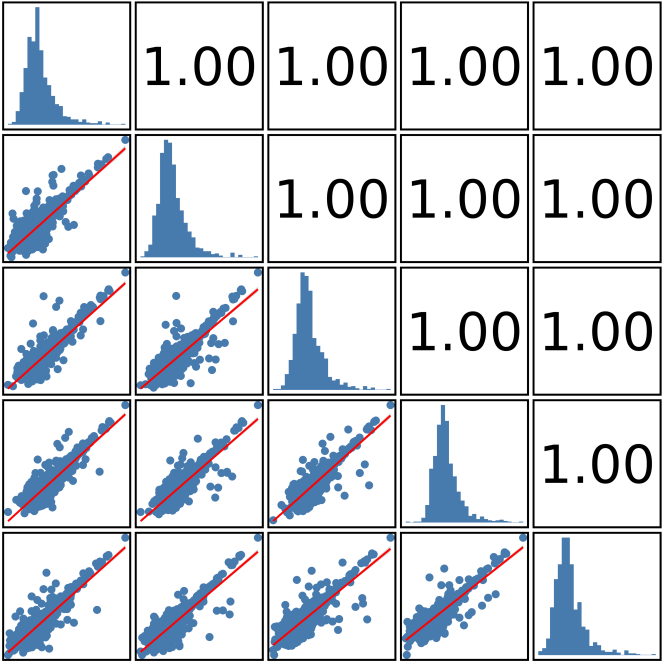


Fig. S2. Correlation Graph of QC samples.


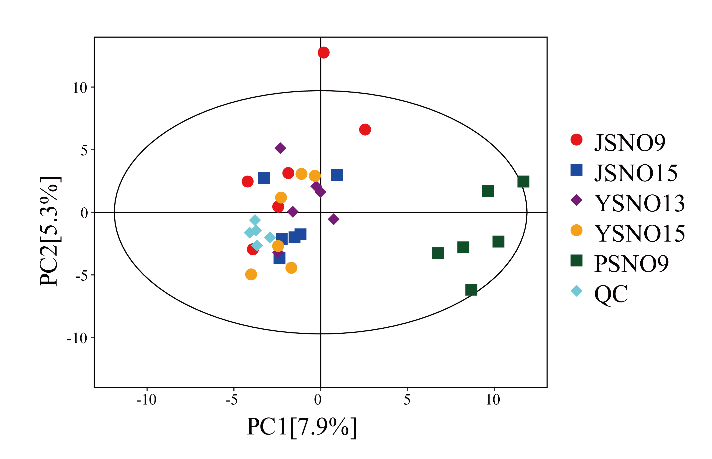


Fig.S3. shows the principal component analysis of five varieties of millet and QC.


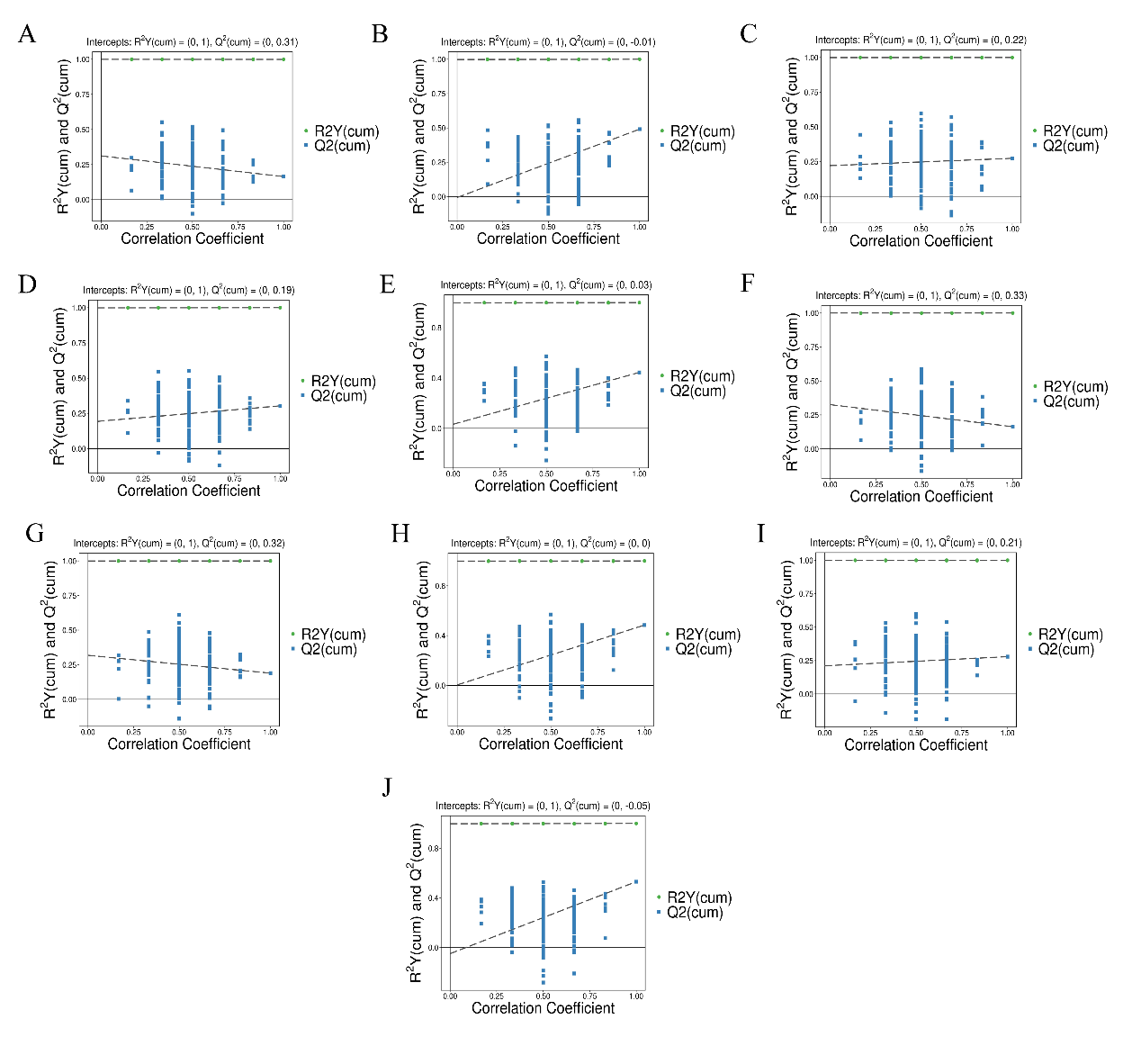


Fig.S4 Quality Control of metabolomics data. (A-J)JSNO9 VS JSNO15, JSNO9 VS SPSNO9, JSNO9 VS YSNO13, JSNO9 VS YSNO16, JSNO15 VS SPSNO9, JSNO15 VS YSNO13, JSNO15 vs The OPLS-DA score graphs among YSNO16, YSNO13 VSPSNO9, YSNO13 VS YSNO16, and YSNO16 VSPSNO9.

Table S1 Statistics of DAMs between JSNO9 VS JSNO15.

| Number | Compound | Class | *P*-valur | Fold Change | VIP |
| --- | --- | --- | --- | --- | --- |
| 1 | (-)-Gallocatechin gallate | flavonoids | 0.005350024 | 1.349911931 | 1.327866916 |
| 2 | Indole-3-acetic acid | phytohormone | 7.26536E-05 | -11.25651432 | 6.206013051 |
| 3 | N6-isopentenyladenosine | phytohormone | 4.11553E-05 | 1.693890279 | 3.849602388 |
| 4 | trans-Zeatin-riboside | phytohormone | 0.00111908 | 1.092954035 | 1.977939351 |
| 5 | L-Ornithine | Amino acid and derivatives;amino acids | 0.000227673 | 0.933341438 | 1.341555195 |
| 6 | Fumaric acid | Organic acids and derivatives | 0.001658528 | -0.702005991 | 1.317416793 |
| 7 | Azelaic Acid | Organic acids | 0.048659991 | -0.485718457 | 1.932620224 |
| 8 | Gallic acid | Phenols | 0.037345044 | -0.440800607 | 1.365451108 |
| 9 | Panaxynol | Miscellaneous | 0.008802046 | -0.976707087 | 1.137698516 |
| 10 | Eriobofuran | Phenols | 0.010840613 | -0.50657673 | 1.76191189 |
| 11 | 5-oxoproline | Amino acid and derivatives | 0.00050802 | 1.306166239 | 1.389296686 |
| 12 | N-Methyltyramine | Benzene and substituted derivatives | 0.037010382 | 0.900003748 | 1.185630164 |
| 13 | Pimelic acid | Fatty Acyls | 0.000282062 | 1.363828348 | 1.619460695 |
| 14 | 2-Phenylacetamide | Benzene and substituted derivatives | 0.000427724 | -1.054457574 | 1.075449424 |
| 15 | Palmitoylethanolamide | Carboximidic acids and derivatives | 0.000412259 | 1.15090616 | 1.175454798 |
| 16 | Moracin O | Phenols | 0.004861473 | 0.62649637 | 1.927059161 |
| 17 | Strychnine | Alkaloids | 0.012288477 | 0.692172666 | 2.297333996 |
| 18 | Wighteone | Flavonoids | 0.033903169 | 0.700798466 | 1.35432385 |
| 19 | 5,6-DHET | Fatty Acyls | 0.007848098 | 1.087266968 | 1.20849567 |
| 20 | 8,9-DiHETrE | Fatty Acyls | 0.031626245 | -1.084016527 | 1.102576154 |
| 21 | Guanosine 3',5'-cyclic monophosphate | Nucleotide and its derivates | 0.029042307 | -0.514078424 | 1.373497055 |
| 22 | cis-Gondoic acid | Lipids | 0.017230572 | 1.49022143 | 1.225378308 |
| 23 | Traumatic acid | Fatty Acyls | 0.000942332 | -0.405550767 | 1.257254385 |
| 24 | Glycerophosphocholine | Cholines | 0.002019481 | -0.37681698 | 1.377439976 |
| 25 | Glucose 1-phosphate | Organooxygen compounds | 0.000732806 | 0.81210682 | 1.121584882 |
| 26 | Mannose 6-phosphate | Organooxygen compounds | 0.010295587 | -0.560773555 | 1.029555089 |
| 27 | Physcion 1-O-beta-D-glucoside;Physcion 8-O-beta-D-monoglucoside;Sissotrin | Anthraquinones;Flavonoids | 0.045429953 | 0.745970636 | 1.659107739 |
| 28 | Decursinol | Coumarins | 0.027615853 | 1.188368695 | 2.318005477 |
| 29 | Artemisinin | Sesquiterpenoids | 0.000962398 | -0.571644853 | 1.07669911 |
| 30 | Neocnidilide | Lactones | 0.029619241 | -1.379326532 | 1.055065405 |
| 31 | Theobromine | Alkaloids | 0.021908306 | -1.187006557 | 1.030124427 |
| 32 | 2-Hydroxy-3-(4-hydroxyphenyl)propenoic acid | Benzene and substituted derivatives | 0.03511403 | -2.123593982 | 1.044188141 |
| 33 | 18alpha-Glycyrrhetinic acid | Triterpenoids | 0.033476455 | 0.847198376 | 1.821182883 |
| 34 | Glutathione | Amino acid and derivatives | 0.023907843 | 1.000438417 | 1.983379659 |
| 35 | L-Pipecolic acid | Amino acid and derivatives | 0.001628139 | 1.683753266 | 1.344233698 |
| 36 | Lincomycin | Carboxylic acids and derivatives | 9.47148E-05 | -1.101015479 | 2.111281446 |
| 37 | Curcumin | Phenols | 0.008134145 | -0.386000929 | 2.01430291 |
| 38 | Vanillic acid | Phenols | 0.011895109 | -0.435107755 | 1.64627777 |
| 39 | 2'-O-Methyladenosine | Purine nucleosides | 1.31196E-07 | -0.840186452 | 1.678514493 |
| 40 | 4-Hydroxyphenylacetylglutamic acid |  | 0.000307266 | -0.505437195 | 1.044577024 |
| 41 | N-D-Glucosylarylamine |  | 0.042492337 | -1.134993746 | 1.75441052 |
| 42 | Beta-Tyrosine | Carboxylic acids and derivatives | 0.017664053 | -0.685728525 | 1.391410711 |
| 43 | Fulvine | Alkaloids | 0.003576164 | -0.678726641 | 2.074097215 |
| 44 | Palmitic acid | Lipids | 3.64981E-06 | 0.907271347 | 1.34856644 |
| 45 | 4-Methyl-5-thiazoleethanol | Azoles | 0.008466085 | -0.705233714 | 1.09090947 |
| 46 | Allocryptopine | Alkaloids | 0.026575898 | -2.34693968 | 1.068584785 |
| 47 | Guanosine | Nucleotide and its derivates | 0.030209208 | 0.934755195 | 1.954446066 |
| 48 | Tenulin | Sesquiterpenoids | 0.011724578 | -0.494317486 | 2.165317236 |
| 49 | (+)-Affinisine | Alkaloids | 0.032196744 | 0.615513159 | 1.70793954 |
| 50 | Luvangetin | Coumarins | 0.04136266 | 1.383787932 | 1.493872976 |
| 51 | Baptifoline | Alkaloids | 0.044878028 | 1.138675306 | 1.362644502 |
| 52 | Beta-Carotene | Vitamins | 0.021078095 | -0.691487248 | 1.892212049 |
| 53 | Boldione | Steroids and steroid derivatives | 0.011080343 | -1.26468319 | 1.520543077 |
| 54 | Cinnamyl cinnamate | Phenylpropanoids | 0.002889514 | 0.835485125 | 1.033088148 |
| 55 | Lupanine | Alkaloids | 0.014984532 | -0.563817895 | 2.376518549 |
| 56 | 2-Picolinic acid | Organic acids | 0.005249024 | 1.349911931 | 1.354420038 |
| 57 | 5-(3-Pyridyl)-2-hydroxytetrahydrofuran | Pyridines and derivatives | 0.014775123 | -11.25651432 | 1.664574445 |
| 58 | Oleic acid;Vaccenic acid;Petroselinic acid | Fatty Acyls | 0.000104636 | 1.693890279 | 1.148773526 |

Table S2 Statistics of DAMs between JSNO9 VS YSNO13.

| Number | Compound | Class | *p*-valur | Fold Change | VIP |
| --- | --- | --- | --- | --- | --- |
| 1 | (-)-Gallocatechin gallate | flavonoids | 0.007058777 | 1.349911931 | 2.086978934 |
| 2 | Indole-3-acetic acid | phytohormone | 2.24768E-06 | -11.25651432 | 6.058716025 |
| 3 | N6-isopentenyladenosine | phytohormone | 6.92591E-06 | 1.693890279 | 2.28656223 |
| 4 | trans-Zeatin-riboside | phytohormone | 0.002919444 | 1.092954035 | 1.706368069 |
| 5 | Glycitein | Flavonoids | 0.02405431 | 0.933341438 | 1.516457629 |
| 6 | Dodecanedioic aicd;Dodecanedioic acid | Fatty Acyls | 0.029752645 | -0.702005991 | 1.256666065 |
| 7 | Adenosine | Nucleotide and its derivates | 6.0785E-06 | -0.485718457 | 1.221090738 |
| 8 | Panaxynol | Miscellaneous | 0.006856033 | -0.440800607 | 1.040106438 |
| 9 | Leukoaminochrome | Indoles and derivatives | 0.019740281 | -0.976707087 | 1.805756664 |
| 10 | 2-Phenylacetamide | Benzene and substituted derivatives | 1.16446E-05 | -0.50657673 | 1.240632544 |
| 11 | 3-Dehydroquinate | Alcohols and polyols | 0.022646944 | 1.306166239 | 2.280776032 |
| 12 | Geranylacetate | Monoterpenoids | 0.016272263 | 0.900003748 | 1.291050191 |
| 13 | Moracin O | Phenols | 0.003332681 | 1.363828348 | 1.946726205 |
| 14 | Strychnine | Alkaloids | 0.049785865 | -1.054457574 | 1.768569377 |
| 15 | ent-16beta,17-dihydroxy-9(11)-kauren-19-oic acid | Diterpenoids | 0.047260197 | 1.15090616 | 1.357422093 |
| 16 | Moracin C | Phenols | 0.000210856 | 0.62649637 | 1.341316071 |
| 17 | cis-Gondoic acid | Lipids | 0.043566539 | 0.692172666 | 1.155320139 |
| 18 | 3-Hydroxycoumarin | Coumarins | 0.017398707 | 0.700798466 | 1.212526062 |
| 19 | Nerylacetate | Monoterpenoids | 0.049327844 | 1.087266968 | 3.045576948 |
| 20 | Glycerophosphocholine | Cholines | 7.36208E-05 | -1.084016527 | 1.852595496 |
| 21 | Oleamide | Fatty Acyls | 0.001455656 | -0.514078424 | 1.167806801 |
| 22 | Lutein | Prenol lipids | 0.028711356 | 1.49022143 | 1.825584707 |
| 23 | Artemisinin | Sesquiterpenoids | 7.96258E-05 | -0.405550767 | 1.092106906 |
| 24 | Miltirone | Diterpenoids | 0.000213955 | -0.37681698 | 1.036094984 |
| 25 | (2S,3R,4E)-2-Amino-4-heptadecene-1,3-diol | Alkaloids | 0.010009506 | 0.81210682 | 1.774054431 |
| 26 | Neocnidilide | Lactones | 0.031388059 | -0.560773555 | 1.071184806 |
| 27 | Theophylline | Alkaloids | 0.024436688 | 0.745970636 | 1.36947761 |
| 28 | Methylecgonine | Alkaloids | 0.015833619 | 1.188368695 | 1.853454103 |
| 29 | Desmethylxanthohumol | Chalcones | 0.027694648 | -0.571644853 | 1.076174387 |
| 30 | N1-Methyl-4-pyridone-3-carboxamide | Pyridines and derivatives | 0.049461852 | -1.379326532 | 1.681163841 |
| 31 | L-Pipecolic acid | Amino acid and derivatives | 5.15859E-07 | -1.187006557 | 1.939238329 |
| 32 | Lincomycin | Carboxylic acids and derivatives | 8.02197E-05 | -2.123593982 | 2.609406447 |
| 33 | 1,2,4-Trimethylbenzene | Benzene and substituted derivatives | 0.025282209 | 0.847198376 | 1.708889119 |
| 34 | Glucosamine | Carbohydrates | 0.006733324 | 1.000438417 | 1.660459982 |
| 35 | Fingolimod hydrochloride | Miscellaneous | 0.01181165 | 1.683753266 | 1.6828317 |
| 36 | Sclareol | Diterpenoids | 0.049116195 | -1.101015479 | 3.116625285 |
| 37 | 2'-O-Methyladenosine | Purine nucleosides | 0.00044174 | -0.386000929 | 1.0374961 |
| 38 | 4-Hydroxyphenylacetylglutamic acid |  | 0.00027932 | -0.435107755 | 1.11543764 |
| 39 | Palmitic acid | Lipids | 1.28655E-06 | -0.840186452 | 1.625415902 |
| 40 | Stearic Acid | Fatty Acyls | 0.001540241 | -0.505437195 | 1.16068616 |
| 41 | Taxiphyllin | Phenols | 0.010717125 | -1.134993746 | 1.752205972 |
| 42 | Calystegine A7 | Alkaloids | 0.033875798 | -0.685728525 | 1.252215697 |
| 43 | 7-(4-Hydroxyphenyl)-1-phenyl-4-hepten-3-one | Phenols | 4.88155E-06 | -0.678726641 | 1.445771437 |
| 44 | Baptifoline | Alkaloids | 0.041833531 | 0.907271347 | 1.178727226 |
| 45 | Riddelline | Alkaloids | 0.045796402 | -0.705233714 | 1.463306916 |
| 46 | Acarbose | Alkaloids | 0.008830286 | -2.34693968 | 3.240065384 |
| 47 | Vitamin A | Vitamins | 0.024791937 | 0.934755195 | 1.533690932 |
| 48 | N-Feruloyl putrescine | Phenolamides | 0.01124292 | -0.494317486 | 1.114509829 |
| 49 | (+)-Afzelechin;(-)-Epiafzelechin | Flavonoids | 0.014671025 | 0.615513159 | 1.177875557 |
| 50 | Luteolin-6-C-glucoside;Orientin | Flavonoids | 0.036445105 | 1.383787932 | 1.880930685 |
| 51 | Zeorin;Dammarenediol II | Triterpenoids | 0.044831622 | 1.138675306 | 1.599232681 |
| 52 | Symlandine;Symphytine | Alkaloids | 0.031203406 | -0.691487248 | 1.259052699 |
| 53 | Phloretic acid;Ethylparaben;3-(2-Hydroxyphenyl)propanoic acid | Phenols;Phenylpropanoic acids | 0.026810191 | -1.26468319 | 1.619232488 |
| 54 | Neogrifolin;Grifolin;Ugaxanthone | Phenols;Xanthones | 0.042287622 | 0.835485125 | 1.535831994 |
| 55 | Oleic acid;Vaccenic acid;Petroselinic acid | Fatty Acyls | 0.000159847 | -0.563817895 | 1.283004725 |
| 56 | (-)-Gallocatechin gallate | flavonoids | 0.007058777 | 1.349911931 | 2.086978934 |

Table S3 Statistics of DAMs between JSNO9 VS YSNO15.

| Number | Compound | Class | *p*-valur | Fold Change | VIP |
| --- | --- | --- | --- | --- | --- |
| 1 | (-)-Gallocatechin gallate | flavonoids | 0.000643222 | 0.807041546 | 1.472630674 |
| 2 | N-((-)-jasmonoyl)-S-isoleucine | phytohormone | 0.000485394 | 1.314194262 | 1.932795901 |
| 3 | Isoquercitrin | flavonoids | 0.006364724 | -1.424041464 | 2.042357956 |
| 4 | Indole-3-acetic acid | phytohormone | 0.000229239 | -11.2088608 | 6.059366171 |
| 5 | L-Tyrosine | amino acids | 0.005877105 | 1.045292896 | 1.618736431 |
| 6 | N6-isopentenyladenosine | phytohormone | 3.11886E-06 | 2.220565132 | 2.667496303 |
| 7 | Naringenin | flavonoids | 0.044609455 | -0.762990194 | 1.245029992 |
| 8 | Indolelactic acid | Indoles and derivatives | 0.038335719 | 0.797424744 | 1.546810114 |
| 9 | Glycitein | Flavonoids | 0.018999898 | 1.081848922 | 1.825427977 |
| 10 | Dodecanedioic aicd;Dodecanedioic acid | Fatty Acyls | 0.028106158 | -0.964159827 | 1.444956314 |
| 11 | N-Methyltryptamine | Tryptamine derivatives | 0.026764709 | -0.971362964 | 1.636471338 |
| 12 | 4-Hydroxyphenyl-2-propionic acid | Phenylpropanoic acids | 0.004759058 | -0.994973027 | 1.756522348 |
| 13 | Sesamol | Phenols | 0.029284783 | 0.864758994 | 1.240387853 |
| 14 | Flavone | Flavonoids | 0.013688398 | -0.765538914 | 1.29962504 |
| 15 | N-Acetylmuramate | Organooxygen compounds | 0.007021911 | -1.123248312 | 1.713190377 |
| 16 | Mahanine | Alkaloids | 0.045157639 | 1.068737267 | 1.309320829 |
| 17 | Strychnine | Alkaloids | 0.009973276 | -1.779501395 | 2.252777724 |
| 18 | 5,6-DHET | Fatty Acyls | 1.55867E-05 | 1.15279341 | 1.891465598 |
| 19 | Guanosine 3',5'-cyclic monophosphate | Nucleotide and its derivates | 0.005650148 | 1.313277999 | 1.878676861 |
| 20 | N-Acetyl-5-hydroxytryptamine | Tryptamine derivatives | 0.025738394 | -2.110511057 | 2.965261124 |
| 21 | Nerylacetate | Monoterpenoids | 0.036289413 | -0.567670271 | 1.074678964 |
| 22 | Glycerophosphocholine | Cholines | 1.39673E-05 | -0.551610188 | 1.299793782 |
| 23 | Physcion 1-O-beta-D-glucoside;Physcion 8-O-beta-D-monoglucoside;Sissotrin | Anthraquinones;Flavonoids | 0.03253956 | 1.318660669 | 2.059893716 |
| 24 | Pipermethystine | Alkaloids | 0.008343175 | -0.883650003 | 1.491760562 |
| 25 | Theobromine | Alkaloids | 0.002804602 | 0.657559352 | 1.321524062 |
| 26 | Dictamnine | Alkaloids | 0.030720688 | -1.548920158 | 3.809699184 |
| 27 | Desmethylxanthohumol | Chalcones | 0.006267803 | -0.73867532 | 1.349760723 |
| 28 | Vanillin | Phenols | 0.000681405 | -0.713576391 | 1.420758924 |
| 29 | Curcumin | Phenols | 0.043197707 | -1.255767938 | 1.714553485 |
| 30 | 2-Hydroxyxanthone | Xanthones | 0.044481459 | -0.55979146 | 1.08874322 |
| 31 | g-Strophanthin | Steroids and steroid derivatives | 0.018576454 | 1.568260263 | 2.301094062 |
| 32 | Beta-Tyrosine | Carboxylic acids and derivatives | 0.021291891 | -0.623771487 | 1.151196331 |
| 33 | Palmitic acid | Lipids | 7.48533E-05 | -0.381150935 | 1.062552535 |
| 34 | 4-Methyl-5-thiazoleethanol | Azoles | 0.000591677 | 0.926165669 | 1.61741284 |
| 35 | Taxiphyllin | Phenols | 0.018230531 | -1.0782066 | 1.699846705 |
| 36 | Allocryptopine | Alkaloids | 0.001171658 | 0.768676928 | 1.430937752 |
| 37 | Guanosine | Nucleotide and its derivates | 0.038246367 | -1.124907494 | 2.366187866 |
| 38 | Mesaconitine | Alkaloids | 0.0173305 | 1.710947682 | 2.289828692 |
| 39 | Liriodenine | Alkaloids | 0.003737148 | 1.385374761 | 1.960844382 |
| 40 | Tenulin | Sesquiterpenoids | 0.020236935 | 1.160422825 | 1.846219654 |
| 41 | Yatein | Lignans | 0.013984798 | 1.537985338 | 1.943617105 |
| 42 | 7beta-(3-Ethyl-cis-crotonoyloxy)-1alpha-(2-methylbutyryloxy)-3,14-dehydro-Z-notonipetranone | Sesquiterpenoids | 0.04069261 | -0.60186114 | 1.051713073 |
| 43 | N-Feruloyl putrescine | Phenolamides | 0.015850923 | -0.502782748 | 1.092911514 |
| 44 | Cinnamyl cinnamate | Phenylpropanoids | 0.002311005 | -0.416741631 | 1.034099934 |
| 45 | Pyridoxal 5'-phosphate | Pyridines and derivatives | 0.048039068 | -1.186330662 | 1.758432999 |
| 46 | 2-Picolinic acid | Organic acids | 0.020708793 | 0.587612075 | 1.121158881 |
| 47 | Shikonin | Quinones | 0.001324754 | -2.124413779 | 2.769031183 |
| 48 | 2'-Deoxyinosine-5'-monophosphate | Nucleotide and its derivates | 0.039741734 | 0.849099467 | 1.461588673 |
| 49 | Butin;2'-Hydroxydihydrodaidzein | Flavonoids;Isoflavonoids | 0.00634033 | -0.913950275 | 1.50093596 |
| 50 | Phloretic acid;Ethylparaben;3-(2-Hydroxyphenyl)propanoic acid | Phenols;Phenylpropanoic acids | 0.008015496 | -1.486296871 | 1.910529653 |

Table S4 Statistics of DAMs between JSNO9 VS PSNO9.

| Number | Compound | Class | *p*-valur | Fold Change | VIP |
| --- | --- | --- | --- | --- | --- |
| 1 | (-)-Epicatechin gallate | flavonoids | 0.032558997 | 0.698478643 | 1.049534844 |
| 2 | (-)-Gallocatechin gallate | flavonoids | 0.000389298 | 0.843131349 | 1.381500693 |
| 3 | N-((-)-jasmonoyl)-S-isoleucine | phytohormone | 0.000275322 | 1.501157294 | 1.866101768 |
| 4 | Indole-3-carboxaldehyde | phytohormone | 1.55744E-05 | 13.81741521 | 5.979695671 |
| 5 | Indole-3-acetic acid | phytohormone | 8.40924E-06 | -11.74519166 | 5.51529628 |
| 6 | L-Tyrosine | amino acids | 0.028602735 | 0.812611785 | 1.190926966 |
| 7 | N6-isopentenyladenosine | phytohormone | 0.000258187 | 0.899662871 | 1.415176639 |
| 8 | Baicalin | flavonoids | 0.014236597 | -1.013859222 | 1.597547357 |
| 9 | Schaftoside | flavonoids | 0.046181963 | 0.919434428 | 1.198540505 |
| 10 | Rhoifolin | flavonoids | 0.005531633 | 5.953297533 | 3.776668089 |
| 11 | Diosmin | flavonoids | 0.019409001 | 5.458009427 | 3.170959231 |
| 12 | 4-Nitrophenol | Phenols | 0.031084335 | -1.073908507 | 2.294924287 |
| 13 | Gallic acid | Phenols | 0.001644527 | -1.268651369 | 1.684070845 |
| 14 | 7-Methylxanthine | Nucleotide and its derivates | 0.047968384 | -0.905980211 | 1.392031299 |
| 15 | Indole | Alkaloids | 0.026780739 | 5.906198722 | 2.700932591 |
| 16 | 5-Hydroxymethylfurfural | Carbonyl compounds | 0.033929957 | 0.912866256 | 1.166592008 |
| 17 | Venlafaxine | Phenol ethers | 0.046342083 | 0.76570439 | 1.19152222 |
| 18 | Adenosine | Nucleotide and its derivates | 0.040756922 | 0.629123669 | 1.733737978 |
| 19 | Panaxynol | Miscellaneous | 0.000404472 | -0.565203392 | 1.117987307 |
| 20 | Eriobofuran | Phenols | 0.025656882 | -1.276740054 | 1.532591721 |
| 21 | Guanine | Nucleotide and its derivates | 0.022613463 | 0.951301291 | 1.539660379 |
| 22 | 5-oxoproline | Amino acid and derivatives | 0.00017105 | -0.742574078 | 1.302784536 |
| 23 | Pimelic acid | Fatty Acyls | 2.08595E-05 | -0.695215122 | 1.286555426 |
| 24 | Deethylatrazine | Triazines | 0.004485915 | 14.15297827 | 5.119836953 |
| 25 | Pentadecanoic acid | Fatty Acyls | 0.029373168 | -0.77481339 | 1.125807064 |
| 26 | Palmitoylethanolamide | Carboximidic acids and derivatives | 0.017049194 | 0.759741349 | 1.642737338 |
| 27 | Lathyrol | Diterpenoids | 0.022064072 | 1.055705892 | 1.332487628 |
| 28 | 5,6-DHET | Fatty Acyls | 5.45369E-05 | 0.772155858 | 1.340853954 |
| 29 | Guanosine 3',5'-cyclic monophosphate | Nucleotide and its derivates | 0.002192349 | 1.864045171 | 2.052046701 |
| 30 | Sitostenone | Steroids and steroid derivatives | 0.004401173 | -1.616292201 | 1.943382992 |
| 31 | Astrocasine | Alkaloids | 0.049791484 | 1.220107359 | 1.260451665 |
| 32 | Quercetin 3-O-neohesperidoside | Flavonoids | 0.008517431 | 6.326457977 | 3.933315117 |
| 33 | Glycerophosphocholine | Cholines | 0.000177499 | -0.794162776 | 1.396258664 |
| 34 | Mannose 6-phosphate | Organooxygen compounds | 0.000684845 | -0.542342831 | 1.086467856 |
| 35 | Physcion 1-O-beta-D-glucoside;Physcion 8-O-beta-D-monoglucoside;Sissotrin | Anthraquinones;Flavonoids | 0.038291715 | 1.405541271 | 2.155915932 |
| 36 | Oleamide | Fatty Acyls | 0.001421787 | -0.537305653 | 1.070585005 |
| 37 | Ellipticine | Alkaloids | 0.015658692 | -1.02144684 | 1.523471033 |
| 38 | D-Xylulose | Carbohydrates | 0.042978408 | -0.972771883 | 1.485325522 |
| 39 | Artemisinin | Sesquiterpenoids | 1.24629E-05 | -0.482281439 | 1.08060562 |
| 40 | Methyl caffeate acid | Phenylpropanoids | 0.046493463 | 0.759063414 | 1.368967887 |
| 41 | Kazinol A | Flavonoids | 0.040087491 | 0.755348209 | 1.101052953 |
| 42 | Theobromine | Alkaloids | 0.004522645 | 0.76581036 | 1.237174087 |
| 43 | Theophylline | Alkaloids | 0.044802189 | 0.616255324 | 1.042766481 |
| 44 | Rosmarinine | Alkaloids | 0.015971905 | -1.310578883 | 1.578493451 |
| 45 | Linarin | Flavonoids | 0.010478375 | 4.207991975 | 2.796782707 |
| 46 | Lincomycin | Carboxylic acids and derivatives | 5.82747E-05 | -1.73453777 | 2.092232339 |
| 47 | Calystegine B2 | Alkaloids | 0.046276598 | 1.000389298 | 1.181892055 |
| 48 | Mitraphylline | Alkaloids | 0.015071746 | 0.572494102 | 1.000478828 |
| 49 | Fingolimod hydrochloride | Miscellaneous | 0.011845861 | 1.832462632 | 1.894037031 |
| 50 | Beta-Sitosterol;beta-Sitosterol | Steroids and steroid derivatives | 0.025877136 | 1.171901572 | 1.482135463 |
| 51 | Dihydroconiferyl alcohol | Phenylpropanoids | 0.001384589 | 0.524309571 | 1.052171366 |
| 52 | 4-Hydroxyphenylacetylglutamic acid |  | 1.82436E-05 | -0.499600622 | 1.09291216 |
| 53 | Fulvine | Alkaloids | 0.016395382 | -1.067926415 | 1.521240559 |
| 54 | Palmitic acid | Lipids | 5.6302E-07 | -0.752139804 | 1.373187056 |
| 55 | 4-Methyl-5-thiazoleethanol | Azoles | 1.60011E-05 | 1.89325445 | 2.188936246 |
| 56 | 8-Methylnonenoate | Fatty Acyls | 0.042379733 | 0.971042267 | 1.319321263 |
| 57 | Allocryptopine | Alkaloids | 0.000130104 | 1.073983785 | 1.586065653 |
| 58 | Calystegine A7 | Alkaloids | 0.035008482 | 1.142258852 | 1.250312345 |
| 59 | 7-(4-Hydroxyphenyl)-1-phenyl-4-hepten-3-one | Phenols | 1.1795E-05 | -0.572159569 | 1.178448595 |
| 60 | Primin | Quinones | 0.019016401 | 0.831987647 | 1.187904496 |
| 61 | 1,5,6-Trihydroxyxanthone | Xanthones | 0.00109435 | 1.926038413 | 2.307814835 |
| 62 | Haplopine | Alkaloids | 0.024551846 | 1.4082143 | 2.248046276 |
| 63 | Sugiol | Diterpenoids | 0.012630529 | 1.526393653 | 1.691315645 |
| 64 | Cinnamyl cinnamate | Phenylpropanoids | 1.39095E-05 | 12.83205937 | 5.762195199 |
| 65 | 3-Methylindole | Alkaloids | 0.00051803 | 2.926180479 | 3.85572797 |
| 66 | 2-Picolinic acid | Organic acids | 0.028478068 | 0.752856527 | 1.167933386 |
| 67 | Shikonin | Quinones | 0.023322044 | -1.701960737 | 2.146086886 |
| 68 | Herniarin | Coumarins | 0.023120121 | 0.830535895 | 1.507892406 |
| 69 | 5-(3-Pyridyl)-2-hydroxytetrahydrofuran | Pyridines and derivatives | 0.005731216 | -1.226414854 | 1.608493607 |
| 70 | Symlandine;Symphytine | Alkaloids | 0.000280437 | -1.140562296 | 1.628621956 |
| 71 | Phloretic acid;Ethylparaben;3-(2-Hydroxyphenyl)propanoic acid | Phenols;Phenylpropanoic acids | 0.004781818 | -2.177637565 | 2.099689367 |
| 72 | Decursin;5-Geranoxy-7-methoxycoumarin;Decursinol angelate | Coumarins | 0.030385978 | -0.677881801 | 1.009784608 |
| 73 | Oleic acid;Vaccenic acid;Petroselinic acid | Fatty Acyls | 0.000132986 | -0.522723243 | 1.098325306 |

Table S5 Statistics of DAMs between JSNO15 VS YSNO13.

| Number | Compound | Class | *p*-valur | Fold Change | VIP |
| --- | --- | --- | --- | --- | --- |
| 1 | Gibberellin A7 | phytohormone | 0.037425231 | 0.737889306 | 1.440886848 |
| 2 | N6-isopentenyladenosine | phytohormone | 1.41695E-05 | -2.44416683 | 3.009819404 |
| 3 | Rutin | flavonoids | 0.042928302 | 1.438196441 | 2.55183777 |
| 4 | Fumaric acid | Organic acids and derivatives | 0.001956499 | 0.672046096 | 1.422045235 |
| 5 | Aflatoxin B1 | Coumarins and derivatives | 0.005773102 | -1.294733756 | 1.889561634 |
| 6 | D-(-)-Quinic acid | Quinolines and derivatives | 0.029914842 | 0.831813421 | 1.617118006 |
| 7 | Hematoxylin | Flavonoids | 0.015744197 | -1.194643474 | 1.788420127 |
| 8 | Shikimic acid | Organic acids and derivatives | 0.024873695 | -0.821409642 | 1.464713766 |
| 9 | Enol-phenylpyruvate | Benzene and substituted derivatives | 0.041162039 | 0.458374433 | 1.015916372 |
| 10 | 5-oxoproline | Amino acid and derivatives | 0.000173595 | 0.737044633 | 1.561122501 |
| 11 | Pimelic acid | Fatty Acyls | 0.000380567 | 0.775849826 | 1.601763166 |
| 12 | 3-Dehydroquinate | Alcohols and polyols | 0.024851363 | 1.124448224 | 2.424969382 |
| 13 | Pogostone | Miscellaneous | 0.017159082 | 0.673711077 | 1.405536791 |
| 14 | Pinobanksin | Flavonoids | 0.032688014 | -0.830793592 | 1.470717046 |
| 15 | Picrocrocin | Monoterpenoids | 0.045115756 | -1.487614181 | 1.693911821 |
| 16 | Wighteone | Flavonoids | 0.042439765 | -0.64945648 | 1.299158247 |
| 17 | Moracin C | Phenols | 0.000126833 | 0.46145698 | 1.241966218 |
| 18 | Xanthyletin | Coumarins | 0.011976333 | -1.3590628 | 2.147943874 |
| 19 | 3-Hydroxycoumarin | Coumarins | 0.033983116 | 0.554495763 | 1.075159472 |
| 20 | Baldrinal | Miscellaneous | 0.03533518 | 0.868210718 | 1.776534919 |
| 21 | Prephenate | Keto acids and derivatives | 0.030241247 | 0.845228718 | 1.42409008 |
| 22 | Glycerophosphocholine | Cholines | 0.002163948 | -0.458900461 | 1.172527959 |
| 23 | (2S,3R,4E)-2-Amino-4-heptadecene-1,3-diol | Alkaloids | 0.033512344 | 0.68197462 | 1.837141944 |
| 24 | Pipermethystine | Alkaloids | 0.029370501 | 1.001883716 | 1.63807339 |
| 25 | Pulegone | Monoterpenoids | 0.022213583 | -0.642643979 | 1.229373264 |
| 26 | L-Pipecolic acid | Amino acid and derivatives | 0.000827175 | -0.549718887 | 1.320043081 |
| 27 | Lincomycin | Carboxylic acids and derivatives | 0.000341596 | -0.797654182 | 1.624733673 |
| 28 | Morroniside | Iridoids | 0.042364432 | 1.389793602 | 1.386496507 |
| 29 | Bruceine D | Diterpenoids | 0.036755773 | 1.220868768 | 2.438090176 |
| 30 | Nobiletin | Flavonoids | 0.004551723 | -0.519870186 | 1.234292947 |
| 31 | Loganin | Terpene | 0.021005288 | 2.296009494 | 3.790944833 |
| 32 | Glucosamine | Carbohydrates | 0.016385281 | 1.124388253 | 1.831897264 |
| 33 | Fingolimod hydrochloride | Miscellaneous | 0.001510939 | 1.044155452 | 1.825240931 |
| 34 | Ergothioneine | Carboxylic acids and derivatives | 0.019884325 | 0.707245781 | 1.332674635 |
| 35 | Vasicine | Alkaloids | 0.025675327 | -2.741101747 | 2.434442861 |
| 36 | 2,6-Dimethyl-7-octene-2,3,6-triol | Monoterpenoids | 0.006416115 | 1.154388342 | 1.930031124 |
| 37 | 2'-O-Methyladenosine | Purine nucleosides | 5.86776E-05 | 0.433855593 | 1.216307296 |
| 38 | Furfuryl acetate | Miscellaneous | 0.025770444 | 0.494362744 | 1.074011856 |
| 39 | Allocryptopine | Alkaloids | 0.002764321 | -0.825287969 | 1.569191382 |
| 40 | Calystegine A7 | Alkaloids | 6.5918E-05 | -1.528399038 | 2.30501808 |
| 41 | 7-(4-Hydroxyphenyl)-1-phenyl-4-hepten-3-one | Phenols | 0.0001912 | -0.411525482 | 1.164983736 |
| 42 | trans-3,5-Dimethoxy-4-hydroxy cinnamaldehydee | Phenylpropanoids | 0.008454564 | 0.777871952 | 1.481152455 |
| 43 | Tenulin | Sesquiterpenoids | 0.040629016 | -1.132652816 | 1.965323999 |
| 44 | Beta-Carotene | Vitamins | 0.042254202 | -0.87670089 | 1.68959943 |
| 45 | Vitamin A | Vitamins | 0.026052125 | 0.821658413 | 1.561631389 |
| 46 | Boldione | Steroids and steroid derivatives | 0.019519438 | 0.830954681 | 1.460761972 |
| 47 | Riboflavine | Vitamins | 0.019253671 | -0.849078541 | 1.500751808 |
| 48 | Lupanine | Alkaloids | 0.037072635 | 1.140113177 | 2.02372875 |
| 49 | Dimethylbenzimidazole | Benzimidazoles | 0.033226467 | 0.606850229 | 1.09189081 |
| 50 | Luteolin-6-C-glucoside;Orientin | Flavonoids | 0.025475006 | 1.859293127 | 2.44046857 |
| 51 | Macrocarpal B;Macrocarpal H;Alisol B | Sesquiterpenoids;Triterpenoids | 0.036777519 | -1.301390238 | 1.642663535 |

Table S6 Statistics of DAMs between JSNO15 VS YSNO15.

| Number | Compound | Class | *p*-valur | Fold Change | VIP |
| --- | --- | --- | --- | --- | --- |
| 1 | Gibberellin A7 | phytohormone | 0.03382423 | 0.652172165 | 1.329950459 |
| 2 | N-((-)-jasmonoyl)-S-isoleucine | phytohormone | 0.000368918 | 1.431565983 | 2.183564528 |
| 3 | Indole-3-carboxaldehyde | phytohormone | 0.00048207 | -0.407443451 | 1.14297069 |
| 4 | L-Tyrosine | amino acids | 0.026722547 | 0.793994588 | 1.341789967 |
| 5 | N6-isopentenyladenosine | phytohormone | 0.00263675 | -1.917491977 | 2.579075818 |
| 6 | trans-Zeatin-riboside | phytohormone | 0.017966188 | -0.880840608 | 1.599116153 |
| 7 | Ononin | flavonoids | 0.027754767 | -0.510257607 | 1.117080786 |
| 8 | sinensetin | flavonoids | 0.013752716 | 1.452385644 | 2.0184473 |
| 9 | (-)-3-(3,4-Dihydroxyphenyl)-2-methylalanine | Amino acid and its derivatives | 0.015131689 | 1.388482539 | 2.610975629 |
| 10 | 4-Hydroxyphenyl-2-propionic acid | Phenylpropanoic acids | 0.041766988 | -0.650163669 | 1.339009653 |
| 11 | Adenosine | Nucleotide and its derivates | 0.000219037 | 0.464431493 | 1.287835417 |
| 12 | Pimelic acid | Fatty Acyls | 0.004134302 | 0.487712131 | 1.202107375 |
| 13 | 2-Phenylacetamide | Benzene and substituted derivatives | 0.014598069 | 0.782361716 | 2.054660315 |
| 14 | 1,2,5,6-Tetrahydro-4H-pyrrolo[3,2,1-ij]quinolin-4-one | Quinolines and derivatives | 0.038396043 | 0.845853087 | 1.726872012 |
| 15 | Deethylatrazine | Triazines | 4.93115E-05 | 0.315712708 | 1.049994016 |
| 16 | 2,3-Dihydro-2-phenyl-4H-benzopyran-4-one | Flavonoids | 0.020070234 | -0.918969736 | 1.589666312 |
| 17 | N-Acetylmuramate | Organooxygen compounds | 0.009732997 | -1.076618714 | 1.795875452 |
| 18 | Taraxerol;Pseudotaraxasterol | Triterpenoids | 0.048541009 | 0.535262483 | 1.147275115 |
| 19 | 3-Carbamyl-1-methylpyridinium (1-Methylnicotinamide) | Pyridines and derivatives | 0.032613593 | 0.426110535 | 1.0268317 |
| 20 | 5,6-DHET | Fatty Acyls | 0.02894236 | 0.637073469 | 1.289051721 |
| 21 | S-Lactoylglutathione | Carboxylic acids and derivatives | 0.049003462 | 1.667734933 | 1.940562665 |
| 22 | Maltotriose | Organooxygen compounds | 0.006354369 | 0.540358942 | 1.23315922 |
| 23 | Juglone | Quinones | 0.034432656 | -0.501102205 | 1.121063432 |
| 24 | Capsanthin | Terpene | 0.03323275 | -0.627190387 | 1.191512183 |
| 25 | (-)-Anonaine | Alkaloids | 0.044613225 | 1.21177686 | 1.787428807 |
| 26 | 18alpha-Glycyrrhetinic acid | Triterpenoids | 0.028576723 | -1.359872704 | 1.958301099 |
| 27 | Glycyrrhetinic acid | Triterpenoids | 0.012804289 | -1.182492284 | 2.135173812 |
| 28 | indolin-2-one | Alkaloids | 0.034888974 | -0.689364208 | 1.6891277 |
| 29 | 3,4-Dihydroxyphenylglycol | Phenols | 0.049900883 | -0.507376465 | 1.075144339 |
| 30 | Vanillin | Phenols | 0.001627597 | -1.044079808 | 1.853354306 |
| 31 | Cheilanthifoline | Miscellaneous | 0.037786783 | -1.00572017 | 1.573991103 |
| 32 | L-Pipecolic acid | Amino acid and derivatives | 0.000259119 | 0.868983104 | 1.714175023 |
| 33 | Lincomycin | Carboxylic acids and derivatives | 0.00026629 | 0.86762529 | 1.704471774 |
| 34 | 2-Hydroxyxanthone | Xanthones | 0.008181343 | -1.073969414 | 1.94363234 |
| 35 | 5-Carboxyvanillic acid |  | 0.046328546 | 0.749023818 | 1.595380357 |
| 36 | g-Strophanthin | Steroids and steroid derivatives | 0.046751254 | 1.514838403 | 2.373400428 |
| 37 | Vanillic acid | Phenols | 0.013542728 | -1.08656363 | 1.836736866 |
| 38 | Guaiacol | Phenols | 0.02077553 | 1.410310331 | 3.037935348 |
| 39 | 2'-O-Methyladenosine | Purine nucleosides | 0.033076868 | 0.951266041 | 3.055460903 |
| 40 | gamma-Diasarone | Lignans | 0.041872848 | -1.456681401 | 2.322312027 |
| 41 | Calystegine A7 | Alkaloids | 0.001705555 | -1.053634448 | 1.834723503 |
| 42 | 2-Hydroxypyridine | Alkaloids | 0.021113726 | -0.493086618 | 1.158783921 |
| 43 | 1-Isomangostin hydrate | Xanthones | 0.024297907 | 0.908451871 | 1.525342035 |
| 44 | Beta-Carotene | Vitamins | 0.013774099 | -1.442811968 | 2.066681111 |
| 45 | Ganoderal A | Triterpenoids | 0.026523596 | -1.241073627 | 1.849596155 |
| 46 | 7beta-(3-Ethyl-cis-crotonoyloxy)-1alpha-(2-methylbutyryloxy)-3,14-dehydro-Z-notonipetranone | Sesquiterpenoids | 0.047971515 | -0.568906943 | 1.082639141 |
| 47 | Mimosine | Alkaloids | 0.025803848 | -1.015125998 | 1.5059432 |
| 48 | Sugiol | Diterpenoids | 0.049537005 | -1.085629522 | 1.777225011 |
| 49 | Shikonin | Quinones | 0.018119084 | -1.043552729 | 1.655589144 |
| 50 | Herniarin | Coumarins | 0.013592495 | -0.448257469 | 1.101351128 |
| 51 | Serotonin | Tryptamine derivatives | 0.037928182 | -0.794846331 | 1.378669659 |

Table S7 Statistics of DAMs between JSNO15 VS PSNO9.

| Number | Compound | Class | *p*-valur | Fold Change | VIP |
| --- | --- | --- | --- | --- | --- |
| 1 | Gibberellin A7 | phytohormone | 0.009612651 | 0.930862625 | 1.442892784 |
| 2 | N-((-)-jasmonoyl)-S-isoleucine | phytohormone | 0.00022197 | 1.618529015 | 2.054195874 |
| 3 | Genistein | flavonoids | 0.039857496 | 2.108355664 | 2.365202634 |
| 4 | Indole-3-carboxaldehyde | phytohormone | 9.73067E-06 | 13.64031342 | 6.266882427 |
| 5 | Indole-3-acetic acid | phytohormone | 0.000224427 | -0.761764958 | 1.391123239 |
| 6 | N6-isopentenyladenosine | phytohormone | 2.30034E-07 | -3.238394238 | 3.074308346 |
| 7 | trans-Zeatin-riboside | phytohormone | 0.016232833 | -0.869252151 | 1.363020941 |
| 8 | Ononin | flavonoids | 0.006852176 | -0.596809094 | 1.102104876 |
| 9 | sinensetin | flavonoids | 0.026986633 | 1.299029149 | 1.73785779 |
| 10 | Baicalin | flavonoids | 0.003125534 | -0.911870261 | 1.448613791 |
| 11 | Cynaroside | flavonoids | 0.004608837 | -1.065653246 | 1.59083119 |
| 12 | Rhoifolin | flavonoids | 0.03559371 | 5.529271416 | 3.352879362 |
| 13 | Diosmin | flavonoids | 0.048554242 | 6.287144212 | 4.105196181 |
| 14 | 4-Nitrophenol | Phenols | 0.044530452 | -0.631028031 | 1.033005973 |
| 15 | 7-Methylxanthine | Nucleotide and its derivates | 0.032207997 | -0.945852706 | 1.340450346 |
| 16 | Indole | Alkaloids | 0.026399315 | 5.946734319 | 2.607000095 |
| 17 | Guanidineacetic acid | Amino acid and derivatives | 0.007921928 | 0.909200555 | 1.429583671 |
| 18 | Sesamol | Phenols | 0.04849631 | -0.739842763 | 1.191351694 |
| 19 | Adenosine | Nucleotide and its derivates | 0.005252943 | 0.924507848 | 1.947220112 |
| 20 | Sphondin | Coumarins | 0.032723694 | -0.8519064 | 1.29175362 |
| 21 | Guanine | Nucleotide and its derivates | 0.011712035 | 1.581163117 | 1.519476241 |
| 22 | N-Methyltyramine | Benzene and substituted derivatives | 0.002716741 | 1.056041212 | 1.604913742 |
| 23 | 2-Phenylacetamide | Benzene and substituted derivatives | 5.68608E-07 | 0.809428351 | 1.498614192 |
| 24 | Deethylatrazine | Triazines | 1.76337E-07 | 14.68081576 | 6.50367255 |
| 25 | Perillyl aldehyde | Prenol lipids | 0.020684942 | 0.976696956 | 2.350015693 |
| 26 | Kirenol | Diterpenoids | 0.025788692 | 3.793060908 | 2.48823397 |
| 27 | Picrocrocin | Monoterpenoids | 0.012335082 | -2.250547476 | 1.252951165 |
| 28 | Guanosine 3',5'-cyclic monophosphate | Nucleotide and its derivates | 0.025306887 | 1.046929197 | 1.378286449 |
| 29 | Sitostenone | Steroids and steroid derivatives | 0.015440352 | -1.278042361 | 1.736856783 |
| 30 | Astrocasine | Alkaloids | 0.015203134 | 1.253900785 | 1.659739629 |
| 31 | Quercetin 3-O-neohesperidoside | Flavonoids | 0.005209291 | 5.3139513 | 3.840517385 |
| 32 | Norbixin | Prenol lipids | 0.023279193 | 0.892735813 | 1.387001579 |
| 33 | Cryptomeridiol 11-rhamnoside | Sesquiterpenoids | 0.016556467 | -1.896329483 | 2.104916352 |
| 34 | DG(16:0/16:0/0:0) | Glycerolipids | 0.005015009 | 1.016965999 | 1.541758152 |
| 35 | Kakuol | Phenols | 0.024684903 | -0.590383285 | 1.062051514 |
| 36 | Mangiferin | Xanthones | 0.048171531 | 1.146050665 | 1.461116713 |
| 37 | Kazinol A | Flavonoids | 0.003184294 | 0.702486943 | 1.243744997 |
| 38 | Cevadine | Alkaloids | 0.039375823 | 1.04372748 | 2.041551764 |
| 39 | Yohimbic acid monohydrate | Alkaloids | 0.040327895 | -1.109492403 | 1.708683331 |
| 40 | Linarin | Flavonoids | 0.00651212 | 5.257047441 | 3.768509647 |
| 41 | Glutathione | Amino acid and derivatives | 0.027539752 | -1.423177834 | 1.756820826 |
| 42 | Vanillin | Phenols | 0.019601081 | -0.772646879 | 1.329051431 |
| 43 | Poncirin | Flavonoids | 0.039969556 | 0.778460851 | 1.151407562 |
| 44 | Nobiletin | Flavonoids | 5.49329E-05 | -0.657594247 | 1.320023074 |
| 45 | Loganin | Terpene | 0.019374299 | 2.330943168 | 3.324745993 |
| 46 | Sclareol | Diterpenoids | 0.000411921 | 1.494478753 | 1.984149842 |
| 47 | Vanillic acid | Phenols | 0.003339966 | -1.179992354 | 1.664929009 |
| 48 | 2,6-Dimethyl-7-octene-2,3,6-triol | Monoterpenoids | 0.009098821 | 1.044953516 | 1.568412203 |
| 49 | 2'-O-Methyladenosine | Purine nucleosides | 6.40466E-08 | 1.10050309 | 1.764416847 |
| 50 | Bellendine | Alkaloids | 0.04939129 | 1.012334542 | 1.614729135 |
| 51 | 4-Methyl-5-thiazoleethanol | Azoles | 0.001929578 | 1.423506698 | 1.916464522 |
| 52 | Rotundine | Alkaloids | 0.048923822 | 1.277897254 | 1.627087845 |
| 53 | Allocryptopine | Alkaloids | 0.022693552 | 0.598636226 | 1.063722303 |
| 54 | Guanosine | Nucleotide and its derivates | 0.018602418 | 1.466014632 | 1.411643659 |
| 55 | Phillyrin;Phillyroside | Phenylpropanoids | 0.043255522 | 2.213337829 | 2.196501727 |
| 56 | 22-Dehydroclerosterol | Steroids | 0.045393617 | 1.051025784 | 1.609910067 |
| 57 | Luvangetin | Coumarins | 0.008566694 | 1.099530786 | 1.482685963 |
| 58 | Putrescine | Phenolamides | 0.014659261 | 3.135405588 | 2.716935577 |
| 59 | 1,5,6-Trihydroxyxanthone | Xanthones | 0.009282 | 1.464827848 | 2.070662117 |
| 60 | Oxoglaucine | Alkaloids | 0.045222976 | -0.663967528 | 1.099329386 |
| 61 | Beta-Carotene | Vitamins | 0.004189952 | -1.869872438 | 2.063192515 |
| 62 | N-Feruloyl putrescine | Phenolamides | 0.047168871 | 1.408787946 | 3.791132667 |
| 63 | Cinnamyl cinnamate | Phenylpropanoids | 1.96509E-06 | 13.22389824 | 6.170318574 |
| 64 | 3-Methylindole | Alkaloids | 0.000198361 | 2.887474122 | 4.071786243 |
| 65 | Riboflavine | Vitamins | 0.039669053 | -0.682465702 | 1.154353601 |
| 66 | 4-Hydroxybenzylamine | Alkaloids | 0.024628694 | 1.031966922 | 1.607357034 |
| 67 | 4-Methylumbelliferone | Coumarins | 0.0339749 | -0.808302628 | 1.160581464 |
| 68 | Symlandine;Symphytine | Alkaloids | 0.001141969 | -0.758373417 | 1.330373997 |
| 69 | Phloretic acid;Ethylparaben;3-(2-Hydroxyphenyl)propanoic acid | Phenols;Phenylpropanoic acids | 0.03823851 | -0.961974113 | 1.191040949 |

Table S8 Statistics of DAMs between YSNO13 VS YSNO15.

| Number | Compound | Class | *p*-valur | Fold Change | VIP |
| --- | --- | --- | --- | --- | --- |
| 1 | (+)-Abscisic acid | phytohormone | 0.006221656 | -0.42872942 | 1.041799701 |
| 2 | N-((-)-jasmonoyl)-S-isoleucine | phytohormone | 0.00021718 | 1.037885441 | 1.730883967 |
| 3 | Indole-3-carboxaldehyde | phytohormone | 4.2043E-05 | -0.399184837 | 1.105562057 |
| 4 | Naringenin | flavonoids | 0.036062216 | -1.887958811 | 4.415886953 |
| 5 | Schaftoside | flavonoids | 0.029935663 | -1.055228576 | 1.842498488 |
| 6 | (-)-3-(3,4-Dihydroxyphenyl)-2-methylalanine | Amino acid and its derivatives | 0.021727132 | 1.223935853 | 2.341513146 |
| 7 | Styrene-cis-2,3-dihydrodiol |  | 0.025273803 | -0.81669846 | 1.356211459 |
| 8 | Sesamol | Phenols | 0.032939141 | 0.68480217 | 1.224510671 |
| 9 | Adenosine | Nucleotide and its derivates | 3.4568E-08 | 0.654765771 | 1.474225457 |
| 10 | 5-oxoproline | Amino acid and derivatives | 0.004108826 | -0.497049346 | 1.126732101 |
| 11 | 2-Phenylacetamide | Benzene and substituted derivatives | 0.00695614 | 0.903900596 | 1.99994145 |
| 12 | N,N-Dimethyl-1,4-phenylenediamine |  | 0.01853577 | 1.768387383 | 2.248985644 |
| 13 | 5,6-DHET | Fatty Acyls | 0.000319849 | 1.026483167 | 1.741001354 |
| 14 | Nicotinamide | Alkaloids | 0.033994155 | -0.613537897 | 1.157774059 |
| 15 | Juglone | Quinones | 0.048750241 | -0.639027315 | 1.267576343 |
| 16 | N-Acetyl-5-hydroxytryptamine | Tryptamine derivatives | 0.010861133 | -9.202206749 | 4.722348547 |
| 17 | Aristolindiquinone | Quinones | 0.030502427 | -0.986611212 | 2.607347449 |
| 18 | Nerylacetate | Monoterpenoids | 0.005368541 | -1.65493724 | 3.393048828 |
| 19 | Glycerophosphocholine | Cholines | 0.000225234 | 0.532406339 | 1.256780339 |
| 20 | Curcolone | Sesquiterpenoids | 0.012488717 | -1.287901412 | 1.810042913 |
| 21 | Abyssinone V | Flavonoids | 0.046671071 | 0.695897809 | 1.226246508 |
| 22 | Capsanthin | Terpene | 0.0347616 | -0.802422594 | 1.373068071 |
| 23 | Encecalin | Phenols | 0.043049264 | 1.027490239 | 2.333075576 |
| 24 | Pipermethystine | Alkaloids | 0.001081045 | -1.147315428 | 1.802792523 |
| 25 | indolin-2-one | Alkaloids | 0.027029988 | -0.548922775 | 1.141786867 |
| 26 | N1-Methyl-4-pyridone-3-carboxamide | Pyridines and derivatives | 0.018603911 | 2.331394234 | 2.31692086 |
| 27 | Vanillin | Phenols | 0.0008948 | -1.010355787 | 1.713778005 |
| 28 | L-Pipecolic acid | Amino acid and derivatives | 1.48442E-07 | 1.418701991 | 2.163485402 |
| 29 | Lincomycin | Carboxylic acids and derivatives | 4.38764E-06 | 1.665279472 | 2.327101672 |
| 30 | Nobiletin | Flavonoids | 0.013871634 | 0.568013419 | 1.172533757 |
| 31 | Loganin | Terpene | 0.033308572 | -2.180144685 | 3.530563305 |
| 32 | 2-Hydroxyxanthone | Xanthones | 0.042063257 | -0.510317871 | 1.020920545 |
| 33 | Benzoin | Stilbenes | 0.035972106 | -0.839636762 | 1.515619249 |
| 34 | 5-Carboxyvanillic acid |  | 0.030608531 | 0.897589045 | 1.567132879 |
| 35 | Glucosamine | Carbohydrates | 0.009105129 | -1.333272819 | 1.926452634 |
| 36 | Fingolimod hydrochloride | Miscellaneous | 4.59955E-05 | -1.211044567 | 1.935215848 |
| 37 | Benzoylagmatine |  | 0.030204691 | -1.082627872 | 2.379984914 |
| 38 | Palmitic acid | Lipids | 0.000123218 | 0.459035517 | 1.174696362 |
| 39 | 4-Methyl-5-thiazoleethanol | Azoles | 0.014557662 | 0.904317502 | 1.477673918 |
| 40 | Schizandrin A | Lignans | 0.031534724 | 0.814255542 | 1.349633341 |
| 41 | Allocryptopine | Alkaloids | 0.00033342 | 1.118617338 | 1.807128914 |
| 42 | Calystegine A7 | Alkaloids | 0.038034538 | 0.47476459 | 1.014135128 |
| 43 | Parsonsine | Alkaloids | 0.038984611 | 1.185536125 | 1.57465974 |
| 44 | 7-(4-Hydroxyphenyl)-1-phenyl-4-hepten-3-one | Phenols | 0.000115586 | 0.590177949 | 1.320198737 |
| 45 | Mesaconitine | Alkaloids | 2.72328E-05 | 2.255302311 | 2.784228079 |
| 46 | Quillaic acid | Triterpenoids | 0.03295054 | 1.258098099 | 1.556676232 |
| 47 | Oxoglaucine | Alkaloids | 0.005017437 | 1.428297521 | 2.17615643 |
| 48 | Riddelline | Alkaloids | 0.022551347 | 0.647219592 | 1.220695272 |
| 49 | Apoatropine | Alkaloids | 0.027771924 | 1.011883132 | 1.376434321 |
| 50 | Acarbose | Alkaloids | 0.006758097 | 2.099684998 | 2.891332084 |
| 51 | 1-Isomangostin hydrate | Xanthones | 0.003472552 | 1.129858847 | 1.777396721 |
| 52 | Allura Red AC | Miscellaneous | 0.016548823 | -1.123102471 | 1.655064174 |
| 53 | Sugiol | Diterpenoids | 0.040675939 | -1.015535353 | 1.435142945 |
| 54 | Swertiaperennin | Xanthones | 0.01826475 | 1.67518632 | 2.02717276 |
| 55 | Shikonin | Quinones | 0.027936085 | -1.079586926 | 1.608471367 |
| 56 | Fustin | Flavonoids | 0.023798942 | -1.060729251 | 1.682339153 |
| 57 | Cinnamyl acetat | Phenols | 0.014615485 | -0.717141752 | 1.343699133 |
| 58 | Dimethylbenzimidazole | Benzimidazoles | 0.021511828 | -0.864292853 | 1.424273586 |
| 59 | Luteolin-6-C-glucoside;Orientin | Flavonoids | 0.013789123 | -1.522200818 | 2.260404184 |
| 60 | 25-Hydroxyvitamin D2-25-glucuronide;25-Hydroxyvitamin D2 25-(beta-glucuronide) | Organooxygen compounds | 0.013794073 | 1.337681751 | 1.82557182 |
| 61 | Secoisolariciresinol monoglucoside;Mascaroside | Lignans;Naphthofurans | 0.044047527 | -2.093965791 | 2.375263901 |

Table S9 Statistics of DAMs between YSNO13 VS PSNO9.

| Number | Compound | Class | *p*-valur | Fold Change | VIP |
| --- | --- | --- | --- | --- | --- |
| 1 | (-)-Epicatechin gallate | flavonoids | 0.003002388 | 0.716835687 | 1.245718357 |
| 2 | N-((-)-jasmonoyl)-S-isoleucine | phytohormone | 9.76071E-05 | 1.224848472 | 1.733522401 |
| 3 | Genistein | flavonoids | 0.021849302 | 1.59754454 | 2.044938053 |
| 4 | Indole-3-carboxaldehyde | phytohormone | 7.49759E-07 | 13.64857204 | 6.099963678 |
| 5 | Indole-3-acetic acid | phytohormone | 0.00087284 | -0.488677336 | 1.060946058 |
| 6 | N6-isopentenyladenosine | phytohormone | 0.000609302 | -0.794227408 | 1.344759074 |
| 7 | trans-Zeatin-riboside | phytohormone | 0.047894033 | -0.66289655 | 1.108463192 |
| 8 | Dihydromyricetin | flavonoids | 0.011743745 | -1.098202301 | 1.653072918 |
| 9 | Rhoifolin | flavonoids | 0.004210506 | 5.493349826 | 3.785892486 |
| 10 | Diosmin | flavonoids | 0.007004732 | 5.441915207 | 3.754645186 |
| 11 | trans-4-Hydroxy-L-proline;4-Hydroxyproline | Amino acid and derivatives;amino acids | 0.026265059 | -0.611507132 | 1.022548078 |
| 12 | Fumaric acid | Organic acids and derivatives | 0.005575163 | -0.490422667 | 1.019203808 |
| 13 | 1H-Indole-3-carboxylic acid;Indole-3-carboxylic | Alkaloids | 0.001458675 | -1.016458734 | 1.482901396 |
| 14 | Hematoxylin | Flavonoids | 0.013799808 | 1.207646272 | 1.536363048 |
| 15 | Gallic acid | Phenols | 0.003521052 | -0.879962378 | 1.377765597 |
| 16 | Shikimic acid | Organic acids and derivatives | 0.034498938 | 0.755842449 | 1.139933137 |
| 17 | L-3-Phenyllactic acid | Phenylpropanoic acids | 0.040670866 | 0.650338796 | 1.389618267 |
| 18 | 5-Hydroxyindole-3-acetic acid | Indoles and derivatives | 0.008489496 | 1.642299429 | 2.010597935 |
| 19 | Adenosine | Nucleotide and its derivates | 0.001700151 | 1.114842127 | 2.013944926 |
| 20 | Guanine | Nucleotide and its derivates | 0.027594147 | 1.286577319 | 1.179296616 |
| 21 | 5-oxoproline | Amino acid and derivatives | 6.31995E-05 | -0.830927457 | 1.429947786 |
| 22 | N-Methyltyramine | Benzene and substituted derivatives | 0.031015713 | 0.757494367 | 1.202931376 |
| 23 | Leukoaminochrome | Indoles and derivatives | 0.000244858 | 1.788156887 | 2.413137701 |
| 24 | Pimelic acid | Fatty Acyls | 2.01167E-05 | -0.622922998 | 1.246109952 |
| 25 | 2-Phenylacetamide | Benzene and substituted derivatives | 1.74455E-08 | 0.93096723 | 1.5793527 |
| 26 | Deethylatrazine | Triazines | 6.11406E-07 | 14.6448649 | 6.318810604 |
| 27 | 3-Dehydroquinate | Alcohols and polyols | 0.032383268 | -1.173851104 | 2.077363093 |
| 28 | Harmaline | Alkaloids | 0.044321066 | -0.960295294 | 1.395198382 |
| 29 | Pinobanksin | Flavonoids | 0.027813331 | 1.072389396 | 1.651658766 |
| 30 | 5,6-DHET | Fatty Acyls | 0.002007793 | 0.645845615 | 1.167489212 |
| 31 | Quercetin 3-O-neohesperidoside | Flavonoids | 0.010522481 | 5.662365353 | 3.798848947 |
| 32 | (E)-3-Hydroxy-5-methoxystilbene | Phenols | 0.017246071 | 0.928731398 | 1.289363656 |
| 33 | D-Xylulose | Carbohydrates | 0.018068988 | -1.068681675 | 1.425598968 |
| 34 | N,N-Dihydroxy-L-phenylalanine |  | 0.03705411 | -1.068320102 | 1.554954678 |
| 35 | Cyclomusalenone | Triterpenoids | 0.042109052 | -1.049494396 | 1.259326578 |
| 36 | Cevadine | Alkaloids | 0.014542423 | 1.043689871 | 2.052124646 |
| 37 | 11-Keto-beta-boswellic acid | Triterpenoids | 0.040549459 | -0.864934863 | 1.212321246 |
| 38 | Linarin | Flavonoids | 0.036146726 | 4.706332911 | 3.390217466 |
| 39 | Vanillin | Phenols | 0.005907668 | -0.738922858 | 1.254105147 |
| 40 | Bruceine D | Diterpenoids | 0.018644064 | -1.224387325 | 2.107005073 |
| 41 | 1,2,4-Trimethylbenzene | Benzene and substituted derivatives | 0.036855693 | -0.781569888 | 1.523836583 |
| 42 | Glucosamine | Carbohydrates | 0.009714698 | -1.203902207 | 1.649018537 |
| 43 | Sparteine | Alkaloids | 0.031010444 | -1.018545857 | 1.305601817 |
| 44 | N-p-Coumaroyl putrescine | Phenolamides | 0.031463631 | 3.456987606 | 2.67608613 |
| 45 | Sclareol | Diterpenoids | 0.000158094 | 1.868395599 | 2.174126091 |
| 46 | Beta-Sitosterol;beta-Sitosterol | Steroids and steroid derivatives | 0.008902286 | 1.075017704 | 1.549001793 |
| 47 | Vasicine | Alkaloids | 0.021118885 | 3.298943932 | 2.446071832 |
| 48 | 2'-O-Methyladenosine | Purine nucleosides | 3.21924E-05 | 0.666647497 | 1.290100014 |
| 49 | 4-Methyl-5-thiazoleethanol | Azoles | 0.000940601 | 1.871406282 | 2.164790245 |
| 50 | Taxiphyllin | Phenols | 0.033520672 | 0.88866817 | 1.361642513 |
| 51 | Allocryptopine | Alkaloids | 8.21426E-05 | 1.423924195 | 1.887861256 |
| 52 | Calystegine A7 | Alkaloids | 5.97884E-05 | 1.827987377 | 2.100739725 |
| 53 | Ecgonine | Alkaloids | 0.009070249 | 0.691169885 | 1.188892542 |
| 54 | Mesaconitine | Alkaloids | 0.007012898 | 0.980857721 | 1.472544376 |
| 55 | Phillyrin;Phillyroside | Phenylpropanoids | 0.048738598 | 1.67204295 | 1.941100589 |
| 56 | trans-3,5-Dimethoxy-4-hydroxy cinnamaldehydee | Phenylpropanoids | 0.014742258 | -0.61808725 | 1.092402174 |
| 57 | 6-Methoxymellein | Benzopyrans | 0.009520507 | -0.839648877 | 1.415760688 |
| 58 | beta-Asarone | Phenylpropanoids | 0.04880439 | 0.999288715 | 1.498660953 |
| 59 | Luvangetin | Coumarins | 0.046000908 | 0.876181031 | 1.153368168 |
| 60 | 1,5,6-Trihydroxyxanthone | Xanthones | 0.020811923 | 1.466023982 | 1.981956736 |
| 61 | Riddelline | Alkaloids | 0.001128213 | 0.978264416 | 1.505339293 |
| 62 | Neoglycyrol | Coumarins | 0.036725444 | -1.232206531 | 1.684577686 |
| 63 | 1-Isomangostin hydrate | Xanthones | 0.009992121 | 0.771150363 | 1.236045879 |
| 64 | Beta-Carotene | Vitamins | 0.036165082 | -0.993171548 | 1.099298863 |
| 65 | Pyridoxine | Vitamins | 0.026525742 | 0.745137641 | 1.155634887 |
| 66 | N-Feruloyl putrescine | Phenolamides | 0.035195021 | 1.490341634 | 3.716643303 |
| 67 | Cinnamyl cinnamate | Phenylpropanoids | 5.29548E-06 | 13.17088363 | 5.990996363 |
| 68 | 3-Methylindole | Alkaloids | 0.003241798 | 2.807884407 | 3.055572998 |
| 69 | Fustin | Flavonoids | 0.008516128 | -1.232987383 | 1.695091353 |
| 70 | Cinnamyl acetat | Phenols | 0.006742015 | -1.210346664 | 1.639410576 |
| 71 | Dimethylbenzimidazole | Benzimidazoles | 0.017059548 | -1.12274884 | 1.445357854 |
| 72 | Deltonin;Gracillin | Steroids and steroid derivatives | 0.016362994 | -0.985495397 | 1.331145795 |
| 73 | Phloretic acid | Phenols | 0.036109713 | -0.912954375 | 1.065682753 |

Table S10 Statistics of DAMs between YSNO15 VS PSNO9.

| Number | Compound | Class | *p*-valur | Fold Change | VIP |
| --- | --- | --- | --- | --- | --- |
| 1 | (-)-Epicatechin gallate | flavonoids | 0.001987195 | 1.168037022 | 1.576205926 |
| 2 | Indole-3-carboxaldehyde | phytohormone | 1.63929E-07 | 14.04775687 | 6.064786472 |
| 3 | Indole-3-acetic acid | phytohormone | 0.006633581 | -0.536330858 | 1.030422842 |
| 4 | N6-isopentenyladenosine | phytohormone | 9.89667E-05 | -1.320902261 | 1.786703357 |
| 5 | Baicalin | flavonoids | 0.011492744 | -0.821609834 | 1.275161379 |
| 6 | Schaftoside | flavonoids | 0.000963647 | 1.459489839 | 1.836691368 |
| 7 | Rhoifolin | flavonoids | 0.006435626 | 5.756106331 | 3.791067315 |
| 8 | Diosmin | flavonoids | 0.0156395 | 6.974277404 | 4.128150044 |
| 9 | (-)-3-(3,4-Dihydroxyphenyl)-2-methylalanine | Amino acid and its derivatives | 0.009306079 | -1.381124418 | 2.183693448 |
| 10 | N-Methyltryptamine | Tryptamine derivatives | 0.009382823 | 1.090571058 | 1.520008504 |
| 11 | Demethoxycapillarisin | Flavonoids | 0.044886485 | -0.938260221 | 1.27434975 |
| 12 | 4-Hydroxyphenyl-2-propionic acid | Phenylpropanoic acids | 0.025612684 | 0.660526766 | 1.132420538 |
| 13 | L-3-Phenyllactic acid | Phenylpropanoic acids | 0.031631969 | 0.822870103 | 1.447351884 |
| 14 | Indole | Alkaloids | 6.06847E-07 | 6.494048933 | 4.128161575 |
| 15 | 5-Aminovaleric acid | Amino acid and derivatives | 0.024861673 | -0.726464752 | 1.11156148 |
| 16 | Toluene-cis-dihydrodiol |  | 0.014205375 | 0.606114671 | 1.041296543 |
| 17 | Sesamol | Phenols | 0.002995625 | -1.088730091 | 1.500501815 |
| 18 | Guanine | Nucleotide and its derivates | 0.004818581 | 1.229341907 | 1.765688823 |
| 19 | Leukoaminochrome | Indoles and derivatives | 0.007462308 | 1.443743916 | 2.162961979 |
| 20 | 1,2,5,6-Tetrahydro-4H-pyrrolo[3,2,1-ij]quinolin-4-one | Quinolines and derivatives | 0.049585132 | -0.873030218 | 1.407993643 |
| 21 | Vasicinone | Alkaloids | 0.042894636 | 1.410558598 | 2.058049151 |
| 22 | Deethylatrazine | Triazines | 4.48876E-08 | 14.36510305 | 6.133069648 |
| 23 | 2,3-Dihydro-2-phenyl-4H-benzopyran-4-one | Flavonoids | 0.035733028 | 0.871686692 | 1.272299286 |
| 24 | 3,4,5-Trimethoxycinnamyl alcohol | Phenylpropanoids | 0.008838175 | -1.020840243 | 1.570211651 |
| 25 | (-)-Carvone | Monoterpenoids | 0.036121321 | 0.712092207 | 1.310283133 |
| 26 | Enoxacin | Diazanaphthalenes | 0.020605005 | 0.74742229 | 1.149247328 |
| 27 | Kirenol | Diterpenoids | 0.02748494 | 3.804515015 | 2.210266004 |
| 28 | 7,8-Dihydroxyflavone | Flavonoids | 0.023603756 | -0.787366646 | 1.155540957 |
| 29 | Juglone | Quinones | 0.012455671 | 0.566795252 | 1.028269669 |
| 30 | Quercetin 3-O-neohesperidoside | Flavonoids | 0.012589035 | 6.136043637 | 3.873141152 |
| 31 | Alpha-Santonin | Sesquiterpenoids | 0.048717769 | 0.949677546 | 1.195091106 |
| 32 | Ellipticine | Alkaloids | 0.000489593 | -1.702013929 | 2.04894207 |
| 33 | Curcolone | Sesquiterpenoids | 0.041734497 | 0.755887499 | 1.048042423 |
| 34 | Abyssinone V | Flavonoids | 0.026880833 | -0.708169928 | 1.151907812 |
| 35 | Cryptomeridiol 11-rhamnoside | Sesquiterpenoids | 0.032131259 | -1.432043983 | 1.512909691 |
| 36 | Pipermethystine | Alkaloids | 0.018126543 | 0.827981469 | 1.287278718 |
| 37 | Yohimbic acid monohydrate | Alkaloids | 0.018967852 | -1.42974662 | 1.957855029 |
| 38 | indolin-2-one | Alkaloids | 0.000397919 | 0.806263987 | 1.33566709 |
| 39 | Linarin | Flavonoids | 0.005482824 | 5.005459488 | 3.12480482 |
| 40 | N1-Methyl-2-pyridone-5-carboxamide | Pyridines and derivatives | 0.031580249 | 1.284267337 | 1.53358448 |
| 41 | N1-Methyl-4-pyridone-3-carboxamide | Pyridines and derivatives | 0.025083458 | -1.353654065 | 1.151424394 |
| 42 | (+)-Camphor | Monoterpenoids | 0.018832768 | 0.696137387 | 1.09625388 |
| 43 | Lincomycin | Carboxylic acids and derivatives | 1.42711E-05 | -1.27622326 | 1.773583139 |
| 44 | Poncirin | Flavonoids | 0.013234612 | 1.512344216 | 1.732167592 |
| 45 | Nobiletin | Flavonoids | 0.00102299 | -0.70573748 | 1.237376445 |
| 46 | Loganin | Terpene | 0.03088935 | 2.21507836 | 3.115852597 |
| 47 | Fingolimod hydrochloride | Miscellaneous | 0.028828492 | 1.359753932 | 1.984362307 |
| 48 | Sclareol | Diterpenoids | 2.13037E-05 | 1.528515042 | 1.951402173 |
| 49 | Guaiacol | Phenols | 0.01939456 | -1.344849686 | 2.514855157 |
| 50 | Xanthurenic acid | Quinolines and derivatives | 0.008230655 | 0.618862431 | 1.10165186 |
| 51 | 2-(2-Hydroxy-2-propyl)-5-methyl-5-vinyltetrahydrofuran | Monoterpenoids | 0.033961897 | -0.683217087 | 1.427579223 |
| 52 | Galanthaminone | Alkaloids | 0.039328917 | 1.147897199 | 1.575336579 |
| 53 | Calystegine A7 | Alkaloids | 0.001166908 | 1.353222786 | 1.714721831 |
| 54 | 7-(4-Hydroxyphenyl)-1-phenyl-4-hepten-3-one | Phenols | 0.000451004 | -0.483610877 | 1.030684969 |
| 55 | Guanosine | Nucleotide and its derivates | 0.009988822 | 1.132026501 | 1.788833916 |
| 56 | 6-Methoxymellein | Benzopyrans | 0.028557897 | -0.661656754 | 1.271815663 |
| 57 | Actinidic acid | Triterpenoids | 0.02298323 | 1.516095944 | 1.75374132 |
| 58 | Luvangetin | Coumarins | 0.027309202 | 1.038926405 | 1.336264509 |
| 59 | Putrescine | Phenolamides | 0.022544716 | 3.881192426 | 2.804454732 |
| 60 | Oxoglaucine | Alkaloids | 0.003327363 | -1.544253365 | 2.003705233 |
| 61 | Cochlearine | Alkaloids | 0.012003401 | -0.829241143 | 1.206458907 |
| 62 | Neoglycyrol | Coumarins | 0.010258961 | -1.798241566 | 2.005888621 |
| 63 | alpha-Peltatin | Lignans | 0.018786182 | 0.984816804 | 1.313971383 |
| 64 | 7beta-(3-Ethyl-cis-crotonoyloxy)-1alpha-(2-methylbutyryloxy)-3,14-dehydro-Z-notonipetranone | Sesquiterpenoids | 0.004017547 | 1.050674515 | 1.463481278 |
| 65 | (-)-Maackiain | Flavonoids | 0.012165683 | 0.954302682 | 1.269142783 |
| 66 | Sugiol | Diterpenoids | 0.009366036 | 1.589595525 | 1.793553239 |
| 67 | N-Feruloyl putrescine | Phenolamides | 0.033698581 | 1.498806896 | 3.657164199 |
| 68 | Cinnamyl cinnamate | Phenylpropanoids | 2.66274E-06 | 13.248801 | 5.888575623 |
| 69 | 3-Methylindole | Alkaloids | 2.21113E-05 | 3.049728081 | 3.947876068 |
| 70 | Herniarin | Coumarins | 0.007405905 | 0.909688199 | 1.57418598 |
| 71 | Serotonin | Tryptamine derivatives | 0.031930584 | 0.974994001 | 1.378272167 |
| 72 | Butin;2'-Hydroxydihydrodaidzein | Flavonoids;Isoflavonoids | 0.009365023 | 0.877378855 | 1.272684105 |
